# Defining peptides in ChEBI

**DOI:** 10.1186/s13321-026-01196-4

**Published:** 2026-05-02

**Authors:** Simon Flügel, Till Mossakowski, Fabian Neuhaus, Erik Pfanenstiel, Martin Glauer, Edgar Haak, Adnan Malik, Noel M. O’Boyle

**Affiliations:** 1 Institute for Computer Science, University of Osnabrück, Neuer Graben 29, Osnabrück, 49074 Lower Saxony Germany; 2 Institute for Cooperating Systems, Otto von Guericke University Magdeburg, Universitätsplatz 2, Magdeburg, 39106 Saxony-Anhalt Germany; 3 Institute of Chemistry, Otto von Guericke University Magdeburg, Universitätsplatz 2, Magdeburg, 39106 Saxony-Anhalt Germany; 4 Chemical Biology Services, EMBL’s European Bioinformatics Institute, Wellcome Genome Campus, Hinxton, Cambridgeshire CB10 1SD UK

**Keywords:** Monadic second-order logic, ChEBI, Peptides

## Abstract

Modern biochemistry is producing vast amounts of chemical knowledge. Ontologies, such as the Chemical Entities of Biological Interest (ChEBI) ontology, can help organising this knowledge. With manual classification alone however, ontologies cannot keep up with the growth of their domain. In this work, we propose a novel taxonomy of 67 classes related to peptides, a large branch in ChEBI with nearly 15,000 compounds. The existing natural language definitions in ChEBI have been expanded and specified more precisely. These natural language definitions are accompanied by a logical axiomatisation in monadic second-order logic (MSOL). To use the axiomatisation for automated classification, a methodology has been developed that translates monadic second-order definitions first into partial first-order definitions and finally into an algorithmic classification. This connects three aspects important to ontological definitions: They reflect the opinions of experts, they are unambiguous, and they can be checked automatically. In our evaluation, we compare the results of our classification to the current taxonomy of ChEBI . This reveals potential inconsistencies in ChEBI as well as areas that might benefit from automated extensions. We also evaluate our natural-language definitions in an expert survey.

**Scientific contribution:** This work provides precise natural-language definitions of 14 current ChEBI classes as well as 53 new peptide-related classes. These definitions are formalised in MSOL and come with an efficient implementation that allows for large-scale molecule classification, including a full classification of ChEBI and PubChem.

## Introduction

Peptides occupy a central role in many biochemical processes, including metabolism, the immune response and the cardiovascular system [1]. Starting with insulin, a wide range of peptides is now being used as drugs, with an increasing number of drugs under development [2, 3]. Current research avenues include antimicrobial peptides [4–6], applications in oncology [7] or peptides as antioxidants [8, 9].

Such a broad field of research requires a common understanding of peptides, as well as means of classifying them and of sharing research results. Ontologies are documents that consist of a vocabulary for a given domain, as well as annotations and a logical theory for the vocabulary that specify the vocabulary’s intended interpretation [10]. Thus, they can help researchers structure their knowledge and build a consensus for central concepts.

In this work, we will focus on one such ontology, the Chemical Entities of Biological Interest (ChEBI) ontology [11], which includes a large selection of peptides: Out of 202,139 classes in version 239 (released 01/2025), 14,925 are subclasses of *peptide*. ChEBI uses two strategies to add new classes: Manual curation by the ChEBI team and annotations by third parties. Following ChEBI ’s rating system, we will refer to the part curated by the ChEBI team as the *3-star subset* and to the rest of ChEBI as the *2-star subset*. In recent years, the 2-star subset has grown faster than the 3-star subset. Currently, it makes up 68% of ChEBI classes (89% of peptides).

Many of the third-party annotations have been created with the support of the rule-based classification system ClassyFire [12]. This is for example done by MetaboLights [13], a repository of metabolomics studies. For MetaboLights, an automated pipeline has been developed to submit new entries to ChEBI . While this speeds up the inclusion of entities into the ontology, it comes with its own challenges: The rules used by the automated classifier might not line up with the intentions of human curators. Furthermore, the rule base has to be updated alongside the ontology when new concepts are added or definitions are revised.

In ChEBI , we can observe that many third-party molecules are classified into more general classes rather than more specific ones: For instance, the *peptide* class has 7423 direct children that are annotated with a Simplified Molecular Input Line Entry System (SMILES) string and thus, most likely, correspond to molecules. Since some of the subclasses of *peptide* form a partition of *peptide* (based on the number of amino acids), we know that all of these direct children should be children of at least one subclass, likely several. The fact that they are not is a limitation for many applications of the ontology. Human users looking for tripeptides might be unable to find the molecules they need because the *tripeptide* class only covers a small fraction of them. Similarly, a machine learning system will suffer from the lack of available data for the subclasses.

Even more problematic are misalignments between the intended definition and the applied definition. This can also happen in human curation, most likely due to imprecise definitions that require an implicit understanding of certain concepts or do not cover specific edge cases. For instance, *oligopeptide* is defined as “A peptide containing a relatively small number of amino acids.” This leaves room for interpretation: Is a molecule with, let’s say, 10 amino acids still small?

In the section "Defining peptides", we propose an alternative taxonomy of 67 peptide-related classes (14 of which are ChEBI classes), including precise natural-language definitions. These definitions are designed to align closely with the intent of the existing ChEBI classes, making implicit assumptions explicit, and to cover edge cases which are not yet formally defined in ChEBI, but arise from the molecules that are currently part of the ontology.

To enable the automatic classification of peptides for this taxonomy, we present in MSOL formalisation section a formalisation of the definitions of the peptide-related classes in monadic second-order logic (MSOL). That this logic is needed to represent the definitions of many chemical classes has been shown previously in [14, 15]. In this work, we argue that the same holds for peptides.

The large expressivity of MSOL limits its computational efficiency. In the section "Implementation", we present how we addressed this concern by implementing several strategies. Firstly, the classification problem is transformed from a theorem proving problem into a model checking problem. Thus, instead of encoding the atoms and bonds of a molecule as axioms, they are represented as a first-order logic (FOL) model. If this model satisfies the definition of a chemical class, then the molecule belongs to the chemical class. Secondly, the monadic second-order definitions are transformed into FOL, and the extensions of those predicates that cannot be defined in FOL are calculated algorithmically. Thirdly, the idea of calculating predicates algorithmically is extended to the whole classification pipeline, eventually replacing the logical definition with a classification algorithm. Each of these steps leads to a significant improvement in performance (the classification algorithm was tested on more than 119 million molecules), while the logical definitions ensure the quality of the result.

The results are evaluated by a comparison with ChEBI (section "Evaluation"). Since our taxonomy differs from ChEBI’s taxonomy of peptides, we compare the density of classifications as well as inter-rater reliability. Further, we asked experts to discuss the classification of 11 molecules where ChEBI and our results contradict each other. However, as we point out in the "Discussion" section, such contradictions are the exception. Overall, we significantly extend the classification depth from 0.12 peptide-related classes per molecule to 0.73 (cf. "Quantitative evaluation). In 97.73% of all cases where new classes are predicted, these are consistent with ChEBI. Thus, we either confirm ChEBI’s classification or extend it in a non-conflicting way.

An online interface for the automatic classification is available [16]. In addition to the classification results, it also explains how a given molecule corresponds to the definitions, justifying the classification.

## Related work

Over the last years, several attempts have been made to use rule-based classification systems in the chemical domain.

### Self-organising ontology

An approach that allows for a high degree of automation is the self-organising ontology proposed by Chepelev et al. [14]. Based on a set of chemicals manually assigned to a certain class, reoccurring features are automatically detected and combined into an Web Ontology Language (OWL) definition. This definition is then tested and iteratively improved. They apply their approach to 60 MeSH and 40 ChEBI classes, which can then be automatically sorted into a hierarchy. With regard to peptides, this method is limited by its assumption that all definitions can be formed from a list of functional groups. However, peptides require a certain configuration of these groups that cannot be expressed in OWL (cf. "MSOL formalisation").

### SODIAC

SODIAC [17] combines SMILES Arbitrary Target Specification (SMARTS) [18] expressions with logical connectors (and, or, not) to formalise classes based on their International Union of Pure and Applied Chemistry (IUPAC) definitions. Out of those classes, an ontology has been constructed containing a total of 3,800 classes, 2,800 of which are defined by SMARTS expressions. The main focus of SODIAC is on natural products and medicinal chemistry.

### ClassyFire

The most comprehensive effort towards a rule-based chemical classification has been conducted by ClassyFire [12]. For ClassyFire, a structure-based taxonomy (ChemOnt) has been developed in which each class has unambiguous formal rules. In total, they cover 4,825 categories. Where it is possible, ChemOnt categories have been mapped to ChEBI classes. At the moment, ClassyFire is actively used in the development of ChEBI to guide manual classification. Similar to our approach, they provide both natural-language descriptions of their classes (albeit without the aspiration to provide a complete definition) and an implementation of their rules that allows classifying arbitrary entities automatically.

The rules used by ClassyFire are based mostly on SMARTS expressions, and, in some cases, on the Markush format [19]. In addition, they use phyisco-chemical properties, chemical formulae and IUPAC names to classify entities. An originally planned open source version has not been realised. In comparison, our rule system relies on a single, more expressive format (MSOL). Also, both our rules and our implementation are open source.

The ChemOnt taxonomy includes entries for peptide, dipeptide, oligopeptide and polypeptide, as well as some other peptide-related classes that are not included in ChEBI. For dipeptide and oligopeptide, their definition explicitly refers to alpha amino acids (instead of arbitrary amino acids), making these classes narrower than their namesakes in ChEBI. This shows that the coverage of ChEBI’s peptide subhierarchy by ChemOnt is very limited. We not only extend the coverage of the peptide subhierarchy significantly, we also add more complex concepts: While a dipeptide consisting of only alpha amino acids can be defined with SMARTS, arbitrary dipeptides are more complex and cannot be expressed with the means used by ClassyFire. Therefore, we have decided to use a more expressive logic that can capture arbitrary amino acid residues. This step is necessary to represent the ChEBI classes which explicitly refer to amino acids other than alpha amino acids.

### FOWL

In [20], a method for assigning FOL annotations to OWL classes has been developed and applied to the ChEBI ontology. The existing SMILES annotations were automatically translated into FOL. The resulting ontology, consisting of FOL and OWL axioms has then been verified by a FOL reasoner.

While this method allows for an automated verification of large parts of the ChEBI , it also has limitations: For one, it relies on the presence of SMILES annotations, which is often not the case. Especially for classes representing groups of molecules (contrary to single molecules), more often than not, no SMILES description is available—as is the case for most classes covered in this paper. In addition, not all of the existing SMILES are meant or able to describe their class accurately. The SMILES language has been developed as a description of single molecules, not for groups of molecules (the corresponding extension for that is SMARTS). While most SMILES used still describe classes accurately through the use of wildcards, in some cases, this does not work. For example, the SMILES specification of the *peptide* class only covers chains of alpha amino acids, not other amino acids or ring structures.

While being less general, our approach uses a stronger logic that can express the concept of peptides completely and provides a better coverage of the peptide domain (most classes defined in this work are not SMILES-annotated). Also, we use expert curation to ensure the correctness of our definition and provide a more efficient classification algorithm than straight-forward reasoning.

### Axiomatisation of symmetric molecules in monadic second-order logic

Kutz et al. [15] have used MSOL to formalise the properties of *fullerenes*, a class of molecules that has a complex 3-dimensional structure. They show that this type of molecules cannot be described in less expressive logics. In order to address concerns regarding the performance of second-order reasoning on the scale of ontologies such as ChEBI, they propose a heterogeneous framework that integrates MSOL reasoning with OWL ontologies. However, the work remains on a theoretical level as no actual classification of molecules is attempted.

In conclusion, most large-scale classification systems to date are either proprietary or do not align their results to existing, manually developed taxonomies. Especially for the domain of peptides, to the best of our knowledge, no comprehensive attempt to formalise the existing hierarchy has been made.

## Defining peptides

In the following, we explain the definitions for peptides and peptide subclasses. We start by describing the development strategy used to produce the definitions (section "Development strategy"). Then, an overview of our revised peptide ontology is given (section "Overview of revised peptide ontology"), showing the formalised classes and their hierarchical relations.

The class definitions themselves are split into 5 sections, based on the classification criterion used: "Classification by charge", "Basic peptide features", which covers intermediate concepts necessary for the "Classification by size" section, followed by "Structure-based classification" and classification by proteinogenic amino acids in "Presence of proteinogenic amino acids".

In this section, for each class, a natural language definition is devised based on the strategy described in the section "Development strategy". The strategy also yields a formalised version of the definitions in MSOL which will be discussed in the section "MSOL formalisation".

### Development strategy

For developing the class definitions, we have integrated several information sources. Our primary source is ChEBI, which provides textual definitions for many classes. In some cases, we have used definitions from the IUPAC Compendium of Chemical Terminology (also called the "Gold Book").

**Fig. 1:**
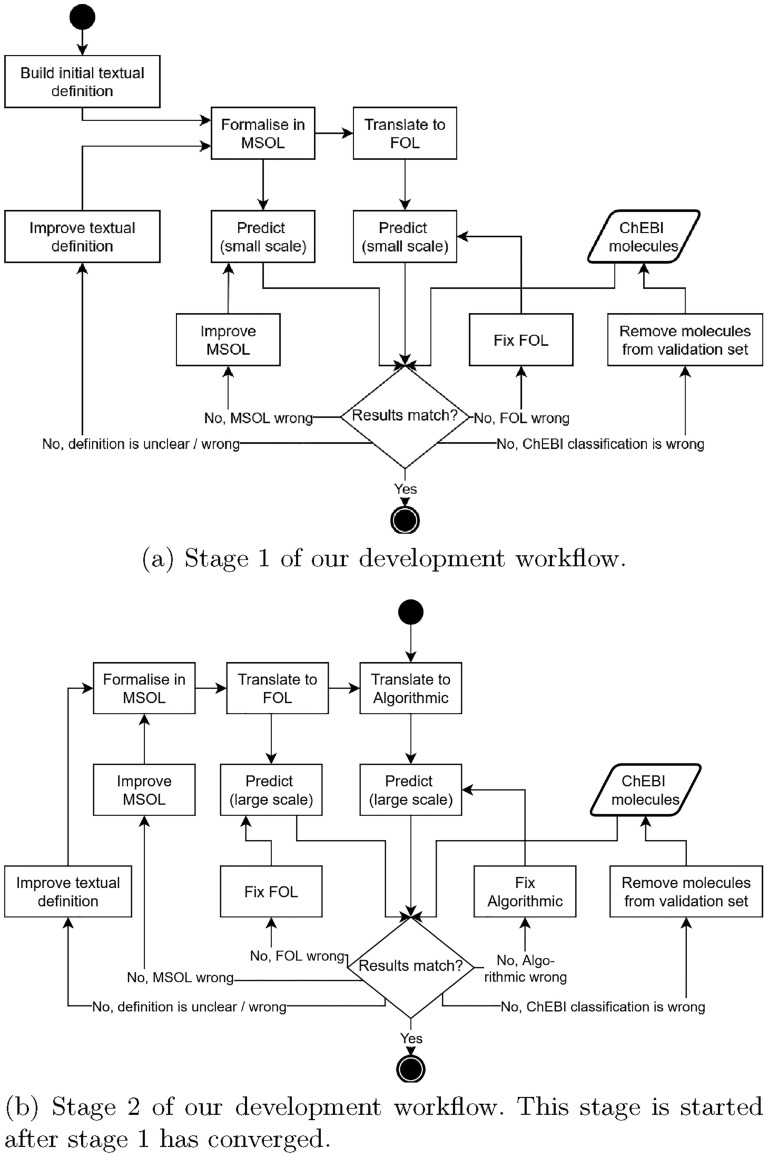
Development workflow for formalising natural language definitions into MSOL and subsequently into FOL and an algorithmic classification

These definitions have been used as the starting point for the workflow described in Fig. 1a. The textual definitions are formalised to MSOL (cf. section 4) and subsequently translated to FOL. The translation process is described in section 5. On a relatively small dataset (in our case, 1,000 peptides from ChEBI), we compare the classification of molecules according to the MSOL formalisation, the version translated to FOL and the original ChEBI label. If the results diverge for a given molecule, we inspect this molecule manually.

Depending on the diagnosed cause of divergence, one of the components of the classification pipeline is adapted. Stage 1 is finished if no samples remain for which we get ambiguous results. The second stage (cf. Fig. 1b) is essentially a repetition of the first stage, but with an important change: Instead of a direct MSOL classification, an algorithmic implementation of the definitions is used. Since the algorithmic implementation is more performant, it can be applied to a larger dataset than in the first stage. Here, we use the 3-star subset of ChEBI with 45,450 molecules to compare the FOL and algorithmic classification against each other as well as against the ChEBI classification.

### Overview of the revised peptide ontology

**Fig. 2:**
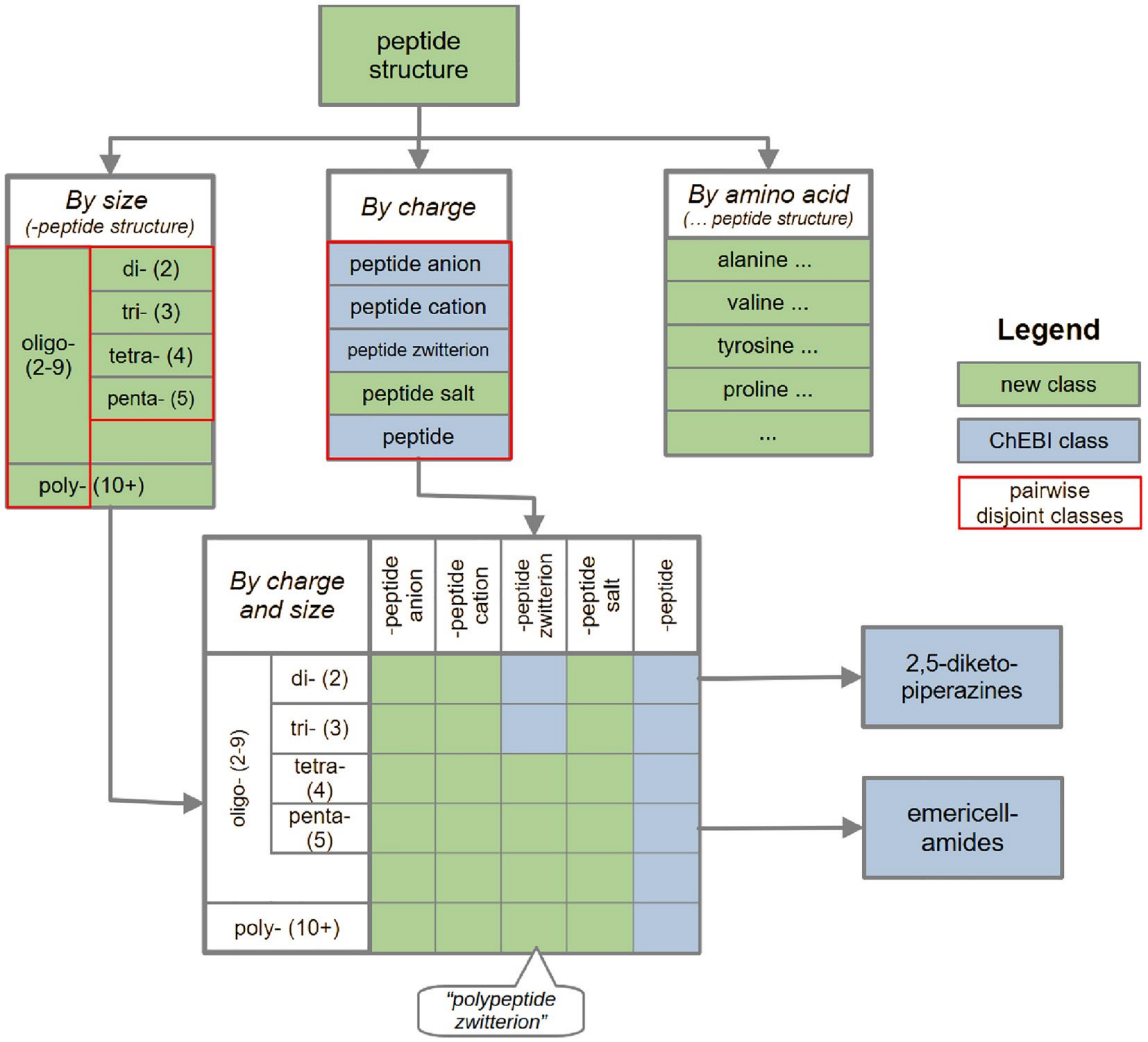
The chemical classes we formalise and their relation to ChEBI classes. Green classes are not part of ChEBI. The classes encircled in red are pairwise disjoint (e.g., a dipeptide structure cannot be a tripeptide structure)

The result of our formalisation is a revised peptide ontology which expands upon the existing ChEBI classes.^1^ The revised ontology is shown schematically in Fig. 2. The full ontology in OWL Manchester syntax can be found in Additional File 1. At the top of the hierarchy, we have added a class *peptide structure* as a common superclass for all classes that follow the same structural rules. This was necessary because the ChEBI classes *peptide anion*, *peptide cation*, *peptide zwitterion* and *peptide* do not have a direct common superclass, despite being closely related. In the revised ontology, these classes are all subclasses of *peptide structure*, alongside *peptide salt*, which has been added because salts do not fit any of the other classes.

A second distinction of *peptide structures* can be made by size, i.e., the number of amino acid residues. ChEBI makes this distinction for the peptide class and in two cases for the peptide zwitterion class. We elevate this distinction by placing it directly under peptide structure, making it independent of the charge-based sub-categorisation of peptide structures. Following ChEBI ’s example, peptide structures are first divided into oligo- and polypeptide structure and then into di-, tri-, tetra- and pentapeptide structures.

Given these two independent subhierarchies of *peptide structure*, we can combine them pairwise to get to some of the classes already included in ChEBI, such as *dipeptide zwitterion*, as well as some new classes. These classes may have further subclasses. As examples, we have included *2,5-diketopiperazines* (which are dipeptides) and *emericellamides* (which are pentapeptides).

In addition to this taxonomy, we also introduce a third set of *peptide structure* subclasses for specific amino acids, namely the 23 proteinogenic amino acids. Since they play an important role in biology, we group peptides which are formed from a specific proteinogenic amino acid under a designated class.

**Fig. 3:**
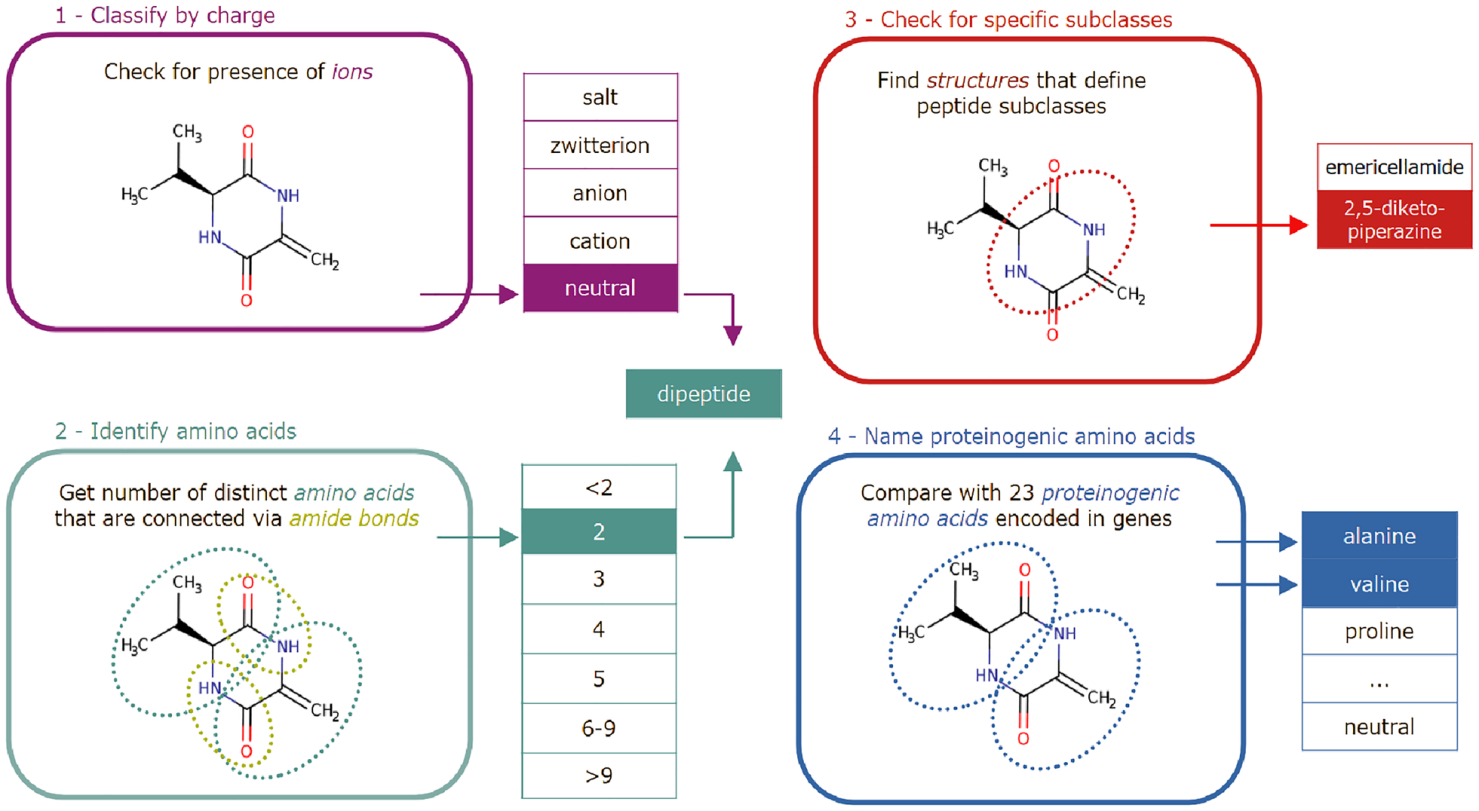
Classification of a (potential) peptide via 4 steps: identification of charges, of amino acid residues, more specific subclasses and proteinogenic amino acids

In summary, to classify a molecule according to the revised ontology, four decisions have to be made (cf. Fig. 3): First, how the molecule is charged (section "Classification by charge"). Second, how many amino acid residues are in a molecule (cf. "Classification by size"). Then, *2,5-diketopiperazines* and *emericellamides* are classified ("Structure-based classification"). Finally, the presence of residues of proteinogenic amino acid has to be verified ("Presence of proteinogenic amino acids").

### Classification by charge

In this section, the charge-based differentiation of peptides is discussed. Note that the definitions in this section are general and not specific to peptides. Instead, we provide the general definitions here (e.g., zwitterion) and derive peptide-specific definitions (e.g., peptide zwitterion) later on.

We distinguish the following categories, following the definitions provided by ChEBI :

#### Definition 1

*(salt - ChEBI)* A salt is an assembly of cations and anions.

#### Definition 2

*(organic anion - ChEBI)* Any organic ion with a net negative charge.

#### Definition 3

*(organic cation - ChEBI )* Any organic ion with a net positive charge.

#### Definition 4

*(zwitterion - ChEBI)* A neutral compound having formal unit electrical charges of opposite sign on non-adjacent atoms. Sometimes referred to as inner salts, dipolar ions (a misnomer).

Note that, by their definitions, these classes are mutually exclusive: Anions have a net negative, cations a net positive, and zwitterions a net neutral charge. Salts can be distinguished from zwitterions by the fact that the charges in the zwitterion have to belong to the same connected component.

A special case in this regard are ylides, dipolar compounds in which an anionic site is directly connected to a positive heteroatom. While ylides are often seen as a subclass of zwitterions [21], ChEBI explicitly excludes charges on adjacent atoms. Therefore, in our classification, ylides are treated as regular peptides and not peptide zwitterions (given that they fulfill the structure-based criteria).

Anions, cations and zwitterions are included because they have peptide-specific subclasses (i.e., *peptide zwitterion*). In addition, salts were added as they neither fit the neutral peptide class, nor any of the other charge-defined classes. This provides a partition of the peptide domain: All *peptide structures* are either anion, cations, zwitterions, salts, or neutral.

### Basic peptide features

Next, we discuss the functional groups that define peptides, i.e., amino groups, carboxylic acids and amides (cf. section "Functional groups"). Based on these groups, we define amino acid residues ("Amino acid residues") and finally peptide structures ("Peptide structure").

Our starting point is the IUPAC definition of peptides, which is also used in nearly identical form by ChEBI: “Amides derived from two or more amino carboxylic acid molecules (the same or different) by formation of a covalent bond from the carbonyl carbon of one to the nitrogen atom of another with formal loss of water. The term is usually applied to structures formed from $\alpha$-amino acids, but it includes those derived from any amino carboxylic acid. ([The side chain] may be any organyl group, commonly but not necessarily one found in natural amino acids).” [22]

While this definition gives us a good understanding of peptides, it is not complete and leaves room for ambiguity. Nevertheless, as it is used by both IUPAC and ChEBI, we will use it as a starting point. Over the course of this section, we will discuss some of the cases where the IUPAC definition does not provide a clear answer and, based on these cases, develop our own definition. In the section "Comparing our definition to IUPAC/ChEBI", we will refer back to the IUPAC definition and compare it to our definition.

We start by discussing the composing features of peptides according to IUPAC . The definition gives us a concise checklist of what to look for in peptides:

- At least 2 amino acid residues.
- A peptide bond, i.e., a covalent bond between a carbonyl carbon of one and the nitrogen atom of another amino acid residue (with formal loss of water).

**Fig. 4:**
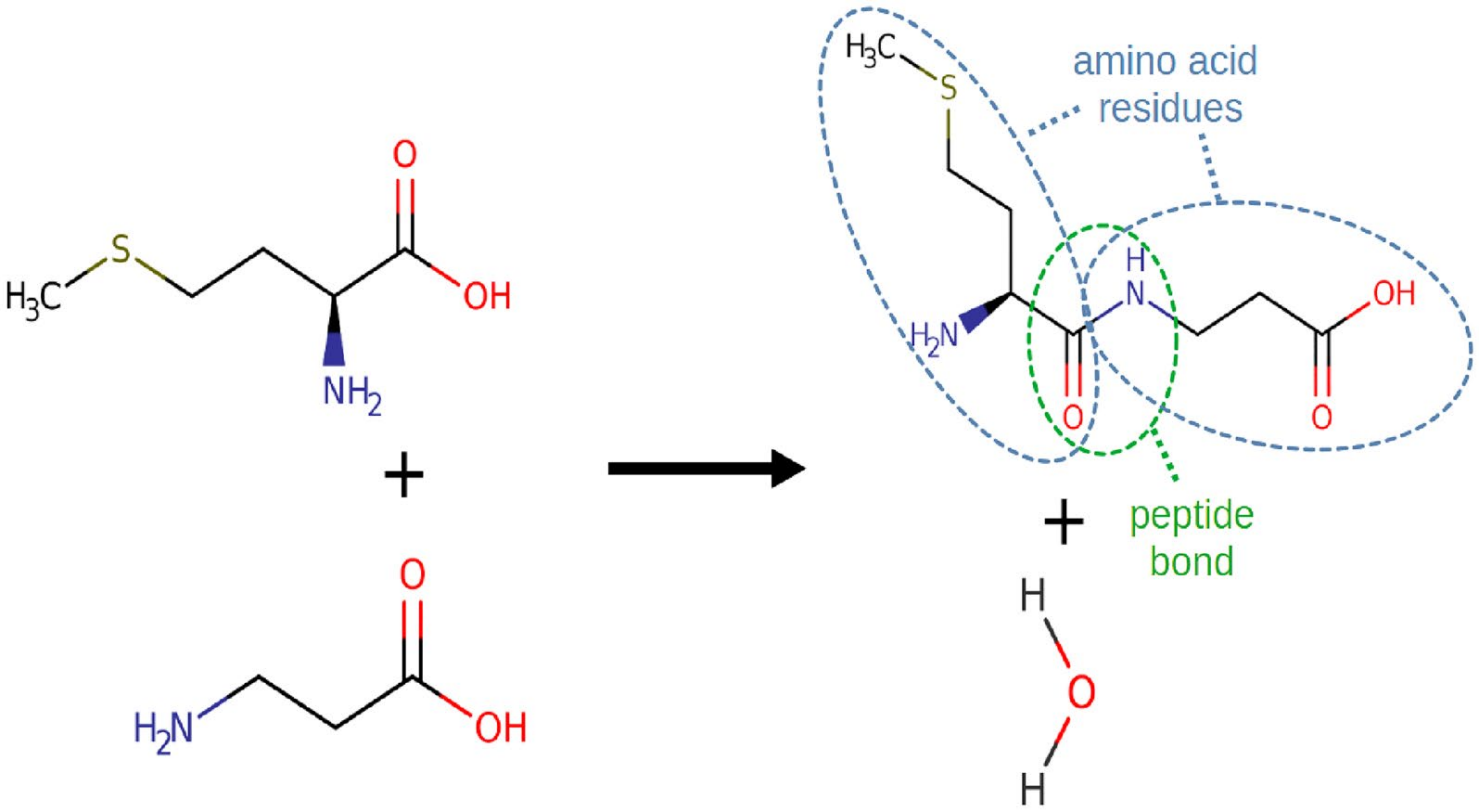
Formation of the dipeptide *Met-*$\beta$*-ala* (CHEBI:74700) from the amino acids *L-methionine* and $\beta$*-alanine.*

To illustrate how a peptide bond looks in practice, we take the example given in Fig. 4. Here, the peptide bond is formed between the carboxyl group of *methionine* and the amino group of $\beta$*-alanine*. The *OH* from the carboxyl group and the *H* from the amino group which do not become part of the peptide form a water molecule. While it is not required for a peptide to be synthesized directly from two amino acids, it has to be derivable in this way on a formal level.

**Fig. 5:**
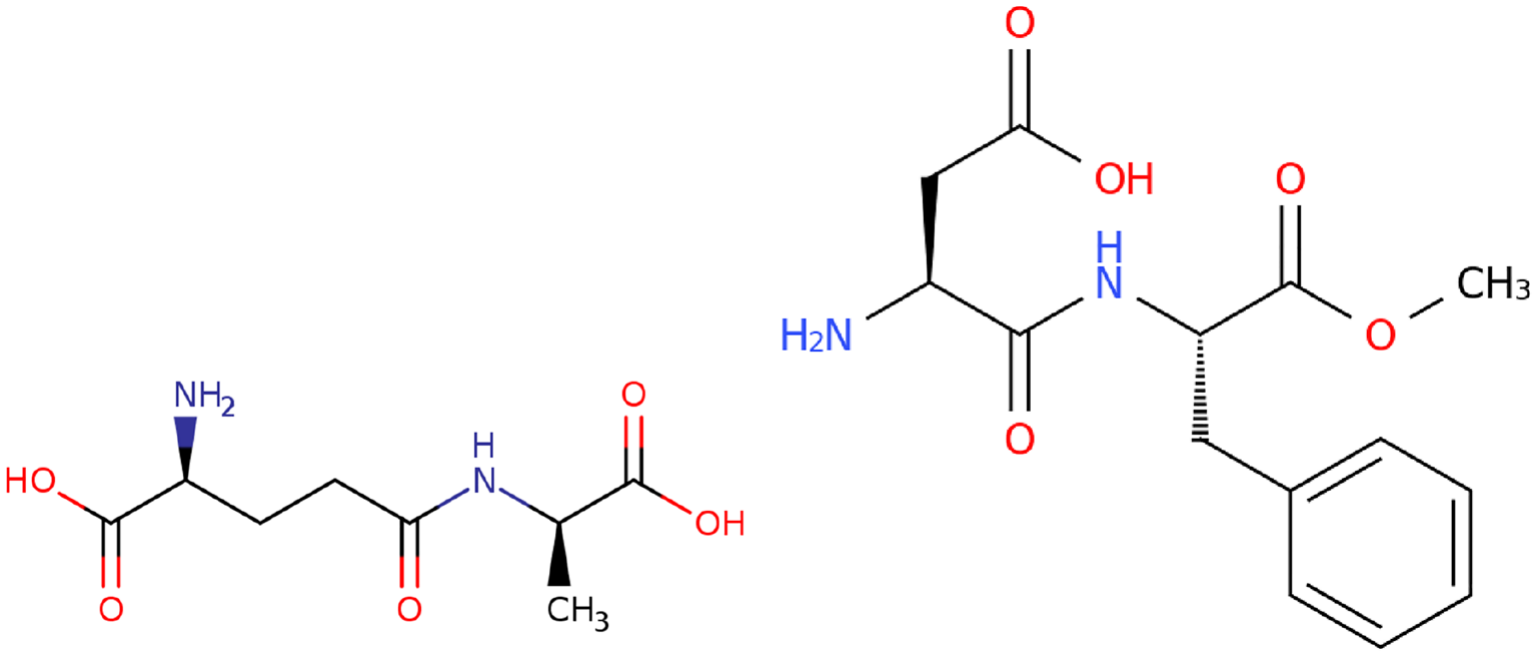
Left: In *L-*$\gamma$*-glutamyl-D-alanine* (CHEBI:16834), the amino acids are connected via an isopeptide bond. Right: *Aspartame* (CHEBI:2877) is derived from *L-aspartic acid* and *methyl L-phenylalaninate*. While the latter is not classified as an amino acid in ChEBI, the resulting structure is considered a dipeptide in ChEBI despite being, strictly speaking, the methyl ester of a dipeptide.

The IUPAC definition also gives advice regarding what it is **not** restricted to:

- $\alpha$-amino acids (e.g., $\beta$*-alanine* in Fig. 4 or any other amino acid is valid as well).
- Side chains from natural amino acids - while there are 23 proteinogenic amino acids that play a special role in biology, the definition includes any type of amino acid. Proteinogenic amino acids will be identified separately (cf. section "Presence of proteinogenic amino acids").
- At which position of the amino acid a peptide bond has been formed (the main carboxy / amino group or a side chain group).

The last point refers to the distinction between the more common *eupeptide* bond, formed between the main carboxy / amino groups of two amino acids, and the less common *isopeptide* bond, in which at least one of the groups is a side chain group. For an example, see Fig. 5 (left). Since glutamyl has two carboxy groups, there are two ways to form peptide bonds: either via the carboxy group at the alpha position or via the carboxy group at the gamma position. The first would be an eupeptide bond, the latter an isopeptide bond. This distinction however is not relevant for our classification.

#### Functional Groups

Let us now take a closer look at the components of peptides, amino acids. In ChEBI, they are defined as follows:

##### Definition 5

*(Amino acid - ChEBI)* A carboxylic acid containing one or more amino groups.

Carboxylic acids in turn are defined in ChEBI as:

##### Definition 6

*(Carboxylic acid - ChEBI)* A carbon oxoacid carrying at least one –C(=O)OH group and having the structure RC(=O)OH, where R is any monovalent functional group.

While this provides us with a clear structural specification for carboxylic acids, in peptides, we also have to consider carboxylic acid derivatives. These are commonly used in place of carboxylic acids by ChEBI and allow for a range of substitutions (e.g., by an ester in *aspartame*, cf. Fig. 5, right). Also, the use of derivatives is clearly intended by ChEBI: In the structural representation for the *peptide* class, the *OH* at the C-terminus is replaced by an *X*, which is described as “X = OH, OR, NH2, NHR, etc.”.

Therefore, we will also allow for *carboxylic acid derivatives* as substitutes for *carboxylic acid groups*, with the following definition:

##### Definition 7

*(Carboxylic acid derivative - ours)* A derivative of a carboxylic acid, having the structure RC(=O)X, where X is a heteroatom.

This definition covers the examples given in ChEBI as well as most other common derivates. Not included are organyl groups such as the pseudohalogen $-CN$ which is theoretically possible as well, but rare in practice due to its reactive nature.

**Fig. 6:**
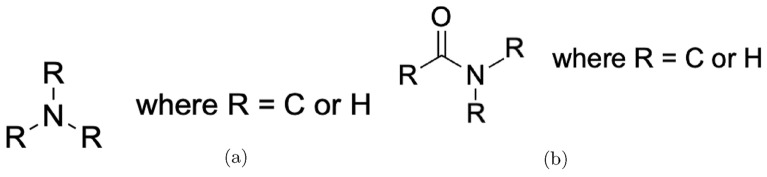
Amino groups and carboxamide groups

Regarding amino groups, we refer to the IUPAC definition [23], which is an extension of the ChEBI definition of *amine* (Fig. 6).

##### Definition 8

*(Amine - IUPAC)* A compound formally derived from ammonia by replacing one, two or three hydrogen atoms by hydrocarbyl groups, and having the general structure RNH2 (primary amines), R2NH (secondary amines) or R3N (tertiary amines).

In other words, amines contain a nitrogen atom bonded to carbon atoms or hydrogen atoms via single bonds. This nitrogen atom constitutes an amino group (cf. Fig. 6a). We do not distinguish between acyl- and arylamines, since ChEBI explicitly includes *aminobenzoic acid* as a 69-membered subclass of *amino acid*. This demonstrates that the term *amine* is clearly intended to encompass both kinds of amines in ChEBI .

Similar to carboxylic acid derivatives, some modifications are possible for the amino group. These include acyl and alkyl substituents (e.g., in *N-formyl-L-methionine*, CHEBI:16552, or in *N,N-diethylglycine*, CHEBI:80633).

Besides amino acids, it is also necessary to identify the bonds that form between the carbonyl carbon of one to the nitrogen atom of another amino acid. The result is usually a carboxamide, with the following definition in ChEBI:

##### Definition 9

*(Carboxamide - ChEBI)* An amide of a carboxylic acid, having the structure $RC(=O)NR_2$. The term is used as a suffix in systematic name formation to denote the $-C(=O)NH_2$ group including its carbon atom.

**Fig. 7:**
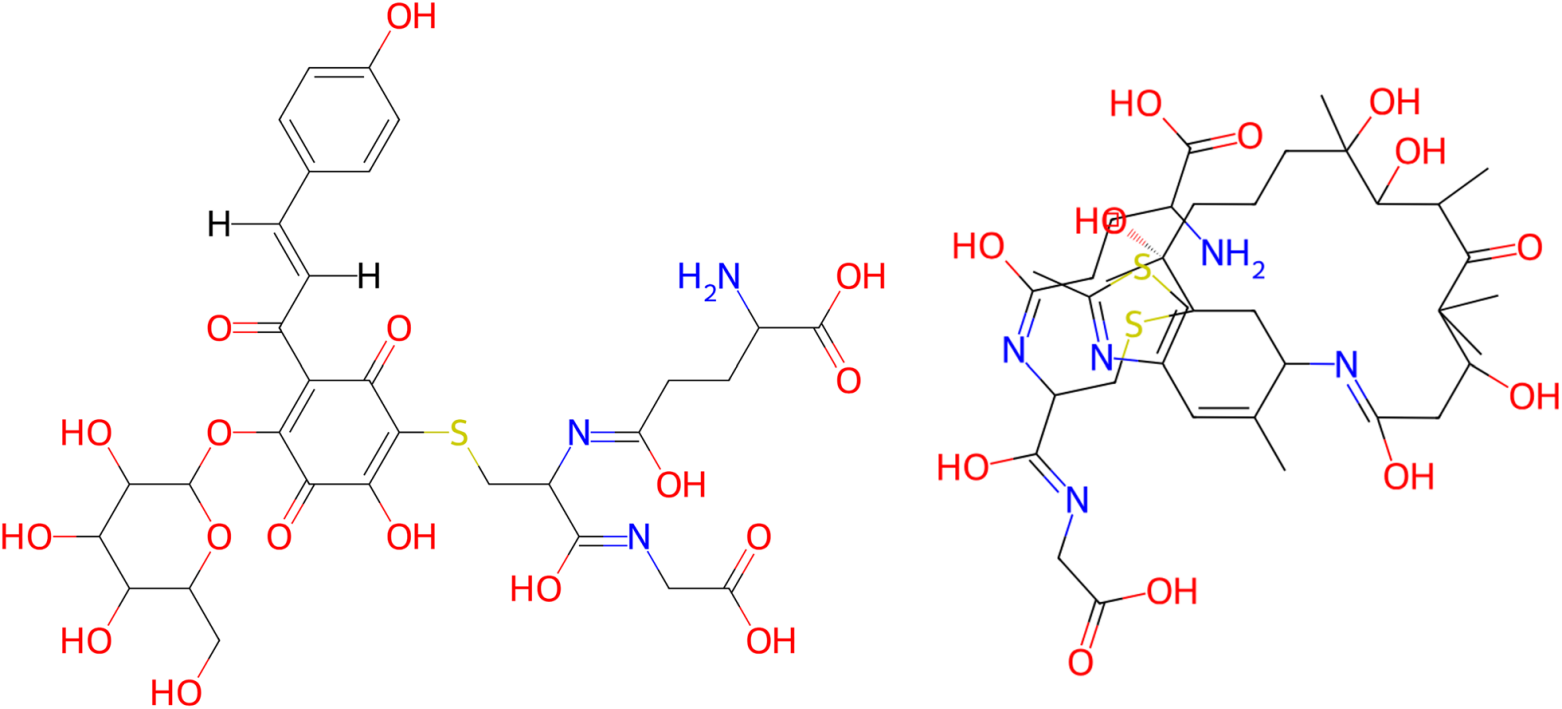
Two examples from ChEBI (CHEBI:194052 and CHEBI:194055), where the imidic acid structure of a peptide bond is used to visualize the molecule. These structures have been submitted to ChEBI by third parties and have not been standardised

**Fig. 8:**
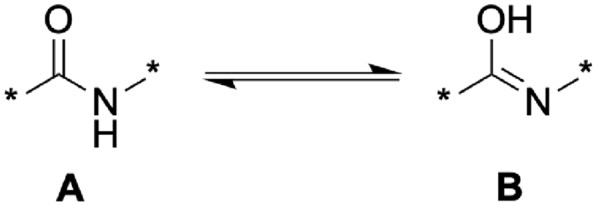
Tautomerisation between the amide form **A** and the imidic acid form **B**

Although the double bond is typically located between the oxygen and carbon atom, ChEBI also includes structures where the double bond is located between the carbon and nitrogen atoms (cf. Fig. 7). This can be explained by the tautomerisation between amides and imidic acids. The rearrangement of the hydrogen atom is visualized in Fig. 8. Both structures can be transformed spontaneously into each other. Therefore, while the depiction in the amidic form is more common, we do not consider structures using the imidic form as belonging to a different class. In the following, we will use the terms *amide bond* and *peptide bond* to refer to both *amides* and *imidic acids*. Formally, ChEBI defines imidic acids as follows:

##### Definition 10

*(Imidic acid - ChEBI)* Compounds derived from oxoacids $R_kE(=O)_l(OH)_m$ ($l \ne 0$) by replacing ${=O}$ by ${=NR}$; thus tautomers of amides. In organic chemistry an unspecified imidic acid is generally a carboximidic acid, $RC(=NR)(OH)$.

#### Amino acid residues

Given these groups, we can define amino acid residues as well as peptides.

As stated earlier, ChEBI defines amino acids as “A carboxylic acid containing one or more amino groups.” While this is certainly correct, we have already seen that modifications of the carboxylic acids and amino groups are allowed in the context of peptides. In addition, the definition raises another question: *How is the number of amino acid residues in a peptide counted?* Or in other words: Where does one amino acid residue stop and another start?

Knowing the exact number of amino acid residues is relevant not only for subclasses such as *tripeptide*, but also decides if a molecule is a peptide at all. Therefore, a general rule is needed that decides which atoms belong to which amino acid residue.

The IUPAC definition of peptides suggests the following strategy: Find the amide bonds and replace them with water, splitting the molecule into unconnected components. Then, check for each part if it fulfils the definition of an amino acid.

**Fig. 9:**
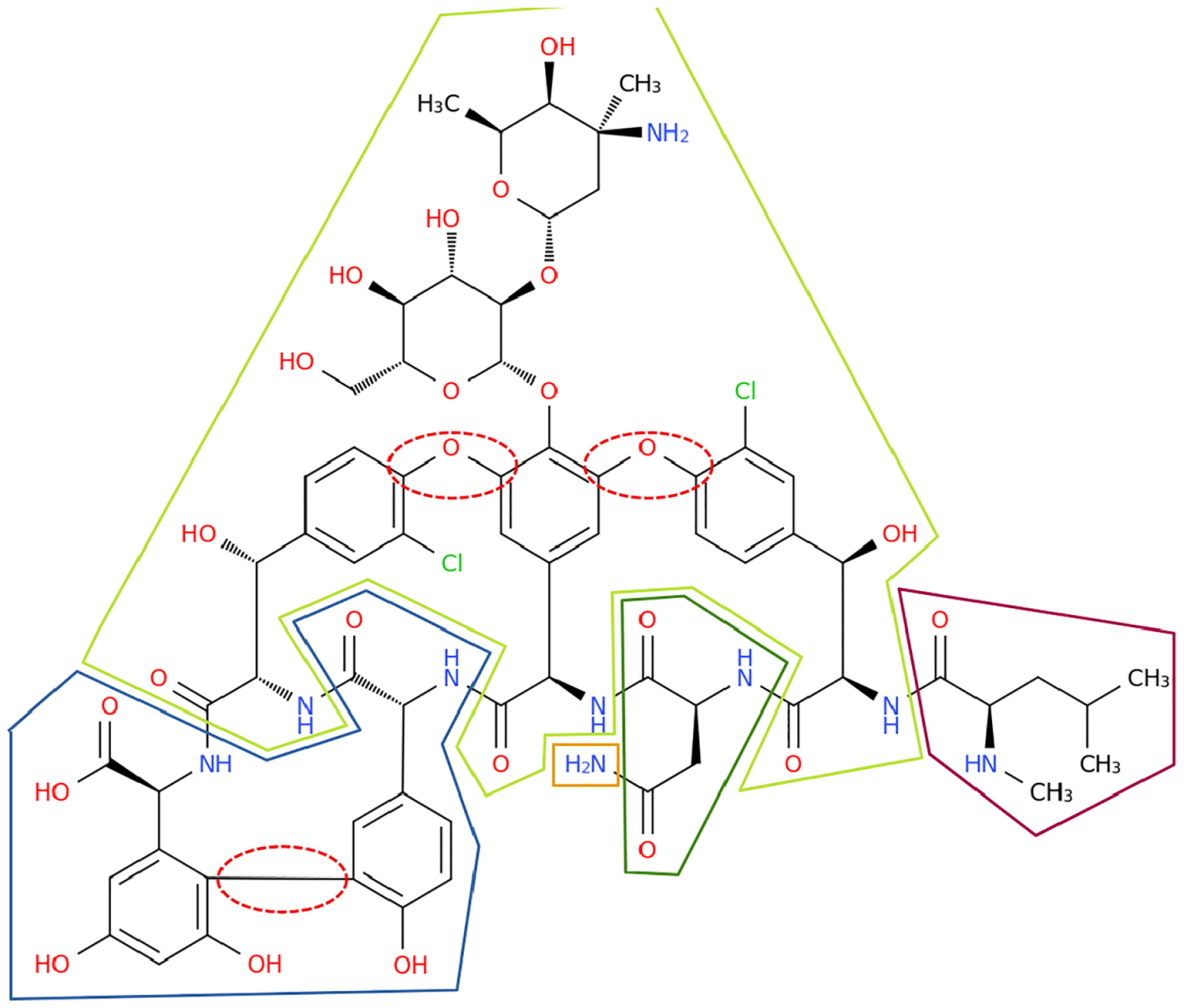
*Vancomycin* (CHEBI:28001) consists of five sections that are separated by amide bonds (highlighted with drawn-through lines), one of which is not an amino acid. In total, the molecule is derived from seven amino acids. The positions of oxidative couplings are shown with dashed lines

In practice, this would lead to some unintuitive results. Take for example the antibiotic *vancomycin* (cf. Fig. 9). Splitting the molecule based on amide bonds only, one gets five sections, four of which have amino groups and carboxylic acids. But calling vancomycin a tetrapeptide would not be an accurate description of the biochemical reality. In fact, *Amycolatopsis orientalis* produces it out of seven amino acids [24]. In three places, aromatic coupling takes place, turning the chain into a cyclic structure.

This illustrates the complexity of defining peptides: The actual derivation of peptides depends on a range of biological and chemical processes. Since this cannot be fully incorporated into general rules for a structure-based classification, an approximation has to be used. The central observation is that amino acids are not linked solely via amide bonds. Often, they form disulfide bridges or aromatic couplings as seen for *vancomycin*. In most cases, these bonds are marked by heteroatoms.

Therefore, we suggest to split molecules at heteroatoms, leaving sections that are connected via a chain of carbon atoms. “Connected via a chain of carbon atoms” means that each section consists of the carbon atoms themselves, as well as the heteroatoms that are attached to it. If a heteroatom neighbours several carbon chains, it may belong to either section. The exception to this are nitrogen atoms of amide bonds - amide bonds can always be clearly cut into two parts. A heteroatom without carbon neighbours forms its own section.

**Fig. 10:**
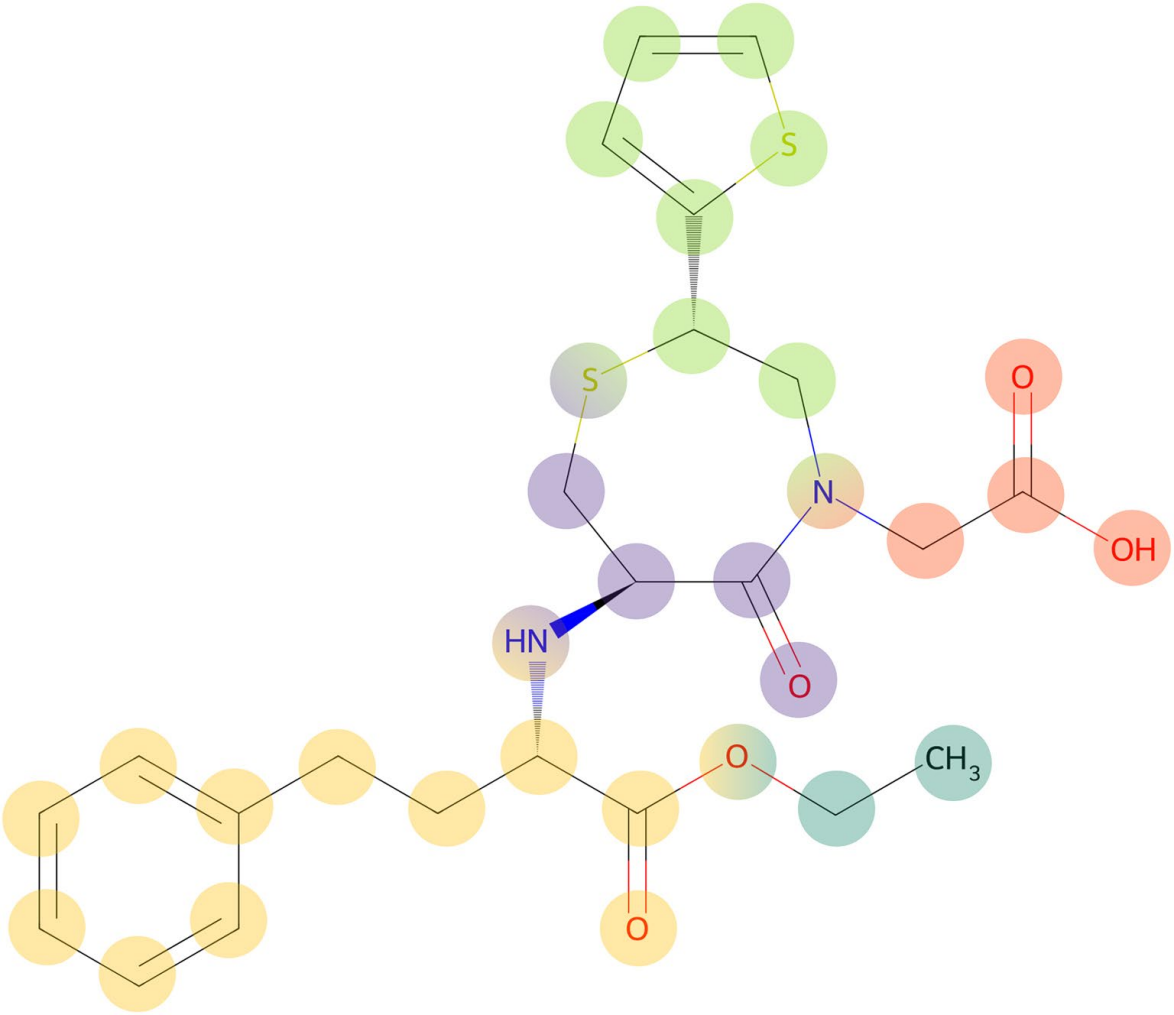
The dipeptide *temocapril* (CHEBI:135771). Sections of the molecule that are connected via a chain of carbon atoms have the same colour. Some heteroatoms belong to several sections

For an example, see *temocapril* (CHEBI:135771, Fig. 10). This molecule has 5 carbon sections that are separated by heteroatoms. The heteroatoms at the border are part of several sections (except for the green and red nitrogen atom, which is not part of the purple section because it is separated from the purple section by an amide bond). Two of the sections (marked in purple and red) constitute amino acid residues. Note that if the sections had not been split up along heteroatoms (and instead only at amide bonds), the purple and red sections would have been directly connected via the green section. This would lead to problems in separating the two amino acid residues.

However, this method is not perfect. For instance, it misses one of the three oxidative couplings in *vancomycin* (cf. Fig. 9). The missed oxidative coupling is a bond between carbon atoms which cannot be differentiated from a regular carbon chain without knowledge of the surroundings and the chemical reactions involved. This demonstrates that a perfect separation of amino acid residues would require a highly complex rule set which is not feasible in this work. Therefore, the “carbon chain rule” is used as a reasonable approximation that works well for most molecules. This leads to the following definition:

##### Definition 11

*(Amino acid residue - ours)* A carboxylic acid or carboxylic acid derivative containing one or more amino groups. The amino groups may carry acyl or alkyl substituents. The residue has to be connected via a chain of carbon atoms which are themselves not part of an amide.

In the section "Presence of proteinogenic amino acids", we will revisit this definition in the context of *proteinogenic* amino acids. There, the side chains of amino acid residues will become relevant for distinguishing different proteinogenic amino acids. These are not covered by the general definition.

#### Peptide structure

With this, we can formulate a definition for *peptide structures*:

##### Definition 12

*(Peptide structure - ours)* (a) Amides derived from two or more amino carboxylic acid molecules or their ions by formation of a covalent bond from *any* carbonyl carbon of one to *any* nitrogen atom of another with formal loss of water (a peptide bond). The term is usually applied to structures formed from $\alpha$-amino acids, but it includes those derived from any amino carboxylic acid. (b) Carboxylic acid groups may be substituted by carboxylic acid derivatives. Amino groups may carry acyl or alkyl substituents. (c) The amino acid residues are delimited by heteroatoms, i.e., exactly the atoms that are connected via a chain of carbon atoms are part of the same amino acid residue. If a heteroatom is connected to different carbon chains, it may be assigned to exactly one of them, excluding those where the adjacent carbon atom is part of an amide.

Paragraph (a) of the definition takes up the IUPAC definition of peptides. The main change we made is the addition of “or their ion” to amino acids since we are explicitly using “peptide structure” as an umbrella term for (neutral) peptides as well as peptide ions (cf. section "Classification by charge"). Also, the term “peptide bond” has been added in order to provide an unambiguous terminology.

In paragraph (b), we refer to the substitutions of functional groups as defined in the section "Functional groups". This covers a range of common substitutions such as halogenations and esterifications in the case of the carboxylic acid, or acetylations and methylations at the amino group.

Paragraph (c) covers the separation of a molecule into different amino acid residues (cf. "Amino acid residues").

#### Comparing our definition to IUPAC/ChEBI

**Table 1:** Comparison of peptide definitions: IUPAC vs. our peptide structure. Sections that only appear in one of the definitions are highlighted

Paragraph	IUPAC (peptide)	Ours (peptide structure)
(a1)	Amides derived from two or more amino carboxylic acid molecules (the same or different)	Amides derived from two or more amino carboxylic acid molecules or their ions
(a2)	by formation of a covalent bond from the carbonyl carbon of one to the nitrogen atom of another with formal loss of water	by formation of a covalent bond from any carbonyl carbon of one to any nitrogen atom of another with formal loss of water (a peptide bond)
(a3)	The term is usually applied to structures formed from $\alpha$-amino acids, but it includes those derived from any amino carboxylic acid	The term is usually applied to structures formed from $\alpha$-amino acids, but it includes those derived from any amino carboxylic acid
(a4)	(R may be any organyl group, commonly but not necessarily one found in natural amino acids)	
(b)		Carboxylic acid groups may be substituted by carboxylic acid derivatives. Amino groups may carry acyl or alkyl substituents
(c)		The amino acid residues are delimited by heteroatoms, i.e., exactly the atoms that are connected via a chain of carbon atoms are part of the same amino acid residue. If a heteroatom is connected to different carbon chains, it may be assigned to exactly one of them, excluding those where the adjacent carbon atom is part of an amide

Having established our own definition of a peptide structure, we will now compare it with the IUPAC definition introduced at the beginning of the section "Basic peptide features". Table 1 gives a side-by-side view of both definitions.

In the beginning of the definition (paragraph a1), we widen the definition by including “or their ions” in addition to amino acids. This is necessary since we do not define the same concept (peptide *structure* instead of peptide). In "Classification by size", a definition of peptides in terms of peptide structures will be provided. The main advantage of first defining an intermediate concept is that this allows us to use the same definition for other related concepts (e.g., peptide zwitterions) as well. The remaining changes in the first sentence (a2) are minor changes for clarification. We do not include section (a4) as it is neither included in the ChEBI definition, nor does it describe a direct feature of peptides. In this context, R refers to the side chains of amino acid residues which we have defined separately (cf. Definition 11).

With the paragraphs (b) and (c), we make three additions to the definition that are meant to clear up ambiguities. First, we introduce the concept of carboxylic acid derivatives (cf. Definition 7). This answers the question: Which modifications of carboxylic acids are valid and which are not? While ChEBI insinuates that there are at least some valid modifications by listing “X = OH, OR, NH2, NHR, etc”, we explicitly say that every heteroatom is a valid replacement for X in the structure $RC(=O)X$.

Examples where this clarification is helpful include *epoxomicin* (CHEBI:42265, X=CC(O)(C)) and *decanoyl-L-Arg-L-Val-L-Lys-L-Arg-chloromethylketone* (CHEBI:156289, X=CCl). An even stronger example are cases where ChEBI provides contradictory examples: *Z-Val-Phe-H* (CHEBI:82818) and *Tyropeptin B* (CHEBI:203965) both have a carboxylic acid derivative with X=H. If we would treat it like a carboxylic acid, we would classify *Z-Val-Phe-H* as a dipeptide (which matches the ChEBI classification), and *Tyropeptin* as a tripeptide (which would not match the ChEBI classification which is dipeptide). If we would not treat it like a carboxylic acid, both molecules would “lose” an amino acid. *Tyropeptin* would be classified as a dipeptide and *Z-Val-Phe-H* as a non-peptide. This shows that no structural rule can explain all molecule classifications in ChEBI at the same time. By choosing one (and automatically applying it to ChEBI , see "Qualitative comparison with ChEBI"), we can identify these inconsistencies and offer a possible resolution.

For amino groups, we also provide a clear rule regarding valid substitutions. Here, acyl or alkyl substituents (and only those) are allowed. While this matches the ChEBI classification in most cases (e.g., for *N-acetyl-L-seryl-L-aspartic acid*, CHEBI:191173), there are also some cases where it does not. For instance, *phosphoramidon* (CHEBI:45363) contains a phosphoramide group. In our classification, this is not a valid substitute for an amino group and therefore, *phosphoramidon* is not considered a peptide. Because this is in contradiction to ChEBI’s classification (dipeptide), we have included *phosphoramidon* in our expert survey (section "Expert survey"). While 2 of 3 experts sided with ChEBI (citing that a hydrolysis is a common biochemical transformation and should therefore be treated the same way as acylations), the third expert agreed with our classification. This goes to show that an unambiguous definition is needed for ontology curation and that leaving these decisions up to the individual curators is a potential source of inconsistencies. We do not claim that our answer in this case is the only correct one. Rather, our definition provides one possible answer which can be adjusted if necessary.

The final source of ambiguity in the definition of peptides lies in the separation of the peptide into individual amino acid residues. Here, the issue is that the IUPAC definition works in the wrong direction: Given two (or more) amino acids, it tells us how to turn it into a peptide. But when deciding if a molecule is a peptide (or differentiating between di- and tripeptides, see "Classification by size"), we have to reverse this step to get to the amino acids. Importantly, we make this reverse steps solely on the structural features of the molecule present, i.e., without background knowledge regarding common amino acids and reaction mechanisms.

To illustrate what this means in practice, we take the example of *methicillin* (CHEBI:6827). This molecule is also part of our expert study (cf. "Expert survey"). The basic approach to classifying this molecule would be to identify the residues of common amino acids (here, cysteine and valine) and possible points for hydrolysis (at the nitrogen and sulfur atoms). However, this leaves the selection of which reactions can be performed where at the molecule to the curator. Instead of relying on this, we suggest a different procedure: Our definition states that “exactly the atoms that are connected via a chain of carbon atoms are part of the same amino acid residue”. This way, we mentally split the molecule at every heteroatom. This is regardless of the plausibility of an actual chemical reaction at that position or if this process leads to residues of common amino acids. In the case of *methicillin*, the result are three parts (separated by nitrogen and sulfur atoms), two of which are amino acid residues. Therefore, we classify *methicillin* as a dipeptide. In this way, we obtain a purely structure-based classification of molecules and a coherent classification scheme. The latter would be much harder to obtain (if at all) with a classification scheme based on possible chemical reactions.

While our classification does not match ChEBI’s classification in some cases (this will be further discussed in the "Evaluation" section), it makes such a classification possible.

### Classification by size

Regarding a size-based classification of peptides, ChEBI distinguishes between *polypeptides* with at least 10 amino acids and *oligopeptides*. In the case of oligopeptides, ChEBI does not give clear instructions regarding the size, instead only defining them as “containing a relatively small number of amino acids.” Still, we can deduce a reasonable range: Since *dipeptide* is a subclass of *oligopeptide*, the lower limit is clearly 2. An upper limit of 9 can be set based on the assumption that *polypeptide* and *oligopeptide* are supposed to be disjoint classes: If a peptide contains “many” amino acids, it cannot contain “few” amino acids at the same time. Aside from *dipeptide* (2 amino acids), ChEBI also includes *tripeptide*, *tetrapeptide* and *pentapeptide* (3, 4 or 5 amino acids) as subclasses of *oligopeptide*.

Usually, an upper bound of 50 to 100 amino acids is applied to peptides as well to distinguish them from proteins. Proteins however are not covered in the scope of ChEBI, which is focused on small molecules.^2^ Therefore, we do not make a distinction between peptides and proteins and use the term *polypeptide* for all structures derived from at least 10 amino acids.

The final classification is a combination of peptide structure and the charge-based classification (cf. section "Classification by charge"). Here, we give two examples of such combinations, the remaining definitions are analogous. *Peptides* are peptide structures with at least 2 amino acids that are neither salts, nor zwitterions, nor anions, nor cations:

#### Definition 13

*(Peptide - ours)* A peptide is a peptide structure of at least 2 amino acids that is neither a salt, nor a zwitterion, anion or cation.

*Tripeptide zwitterions* are peptide structures of exactly 3 amino acids that are zwitterions:

#### Definition 14

*(Tripeptide zwitterion - ours)* A tripeptide zwitterion is a peptide structure of exactly 3 amino acids (a tripeptide structure) that is a zwitterion.

### Structure-based classification

So far, we have defined peptides by their general pattern, with subclassification based on charge and number of amino acids. However, many peptide subclasses are more specific, e.g., *emericellamide* and *2,5-diketopiperazine*.

Emericellamides are defined as:

#### Definition 15

*(emericellamide - ChEBI)* A cyclodepsipeptide derived from an N-($\beta$-hydroxyacyl)glycyl-L-valyl-L-leucyl-L-alanyl-L-alanine by the formal condensation of the alcoholic hydroxy group with the C-terminal carboxy group.

**Fig. 11:**
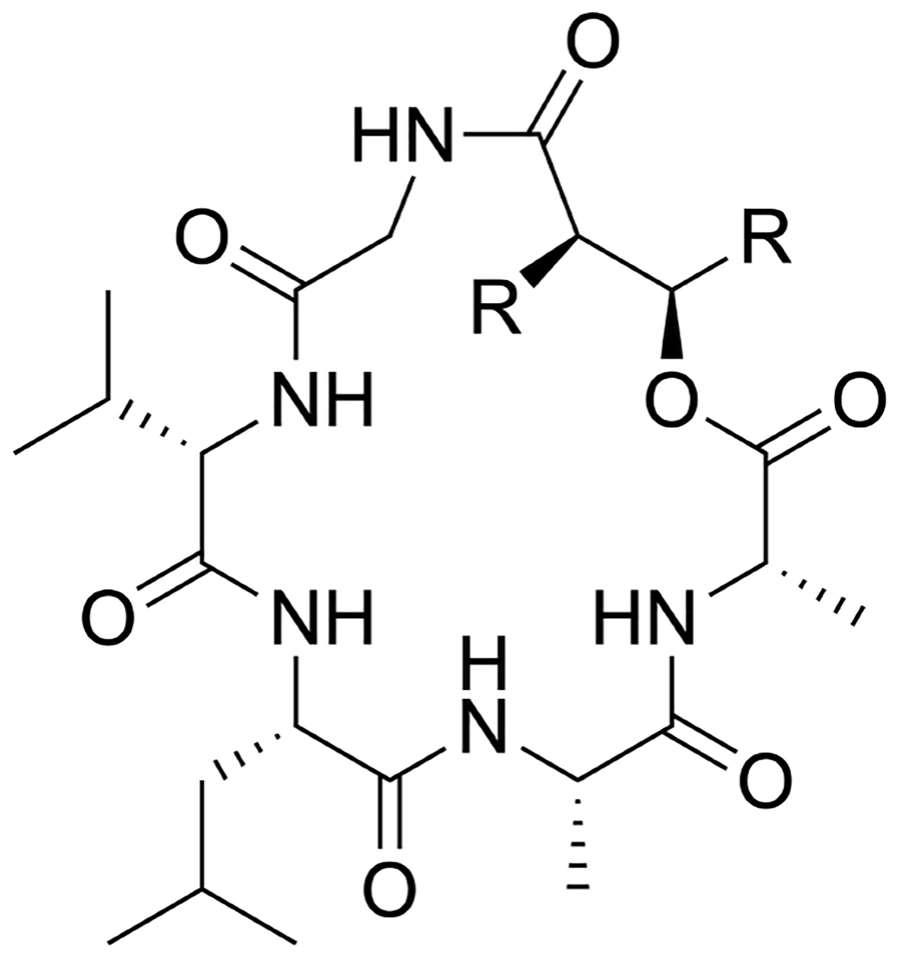
Common substructure for the emericellamide class. The emericellamides are formed by replacing the Rs with hydrocarbyl groups

While the relation is not explicitly given by ChEBI, the 5 amino acids mentioned by the definition suggest that all emericellamides are pentapeptides. Furthermore, there are only a few positions at which additional groups can be added to the ring, namely between the N-terminus and C-terminus (otherwise, the names of the amino acids would change, see Fig. 11).

2,5-diketopiperazines are defined as:

#### Definition 16

*(2,5-diketopiperazines - ChEBI)* Any piperazinone that has a piperazine-2,5-dione skeleton.

The piperazine-2,5-dione skeleton dictates a structure of two amino acids. Here, the degree of freedom is in the side chains, which underlie no restrictions.

### Presence of proteinogenic amino acids

So far, we have not considered distinctions between different amino acids. However, for many applications, it is useful to know which peptides contain residues of which amino acids. Proteinogenic amino acids in particular are relevant in biochemistry. These are also grouped under their own ChEBI class:

#### Definition 17

*(proteinogenic amino acid - ChEBI)* Any of the 23 $\alpha$-amino acids that are precursors to proteins, and are incorporated into proteins during translation. The group includes the 20 amino acids encoded by the nuclear genes of eukaryotes together with selenocysteine, pyrrolysine, and N-formylmethionine. Apart from glycine, which is non-chiral, all have L configuration.

To identify these amino acids in peptides, it is necessary to account for the fact that one does not have access to the original amino acid, but only to its residue. Depending on the peptide bonds formed, one amino acid corresponds to different residues (e.g., depending on whether it is located at the C-, N-terminus or in the middle of a peptide chain). Therefore, for each atom in the amino acid that is not part of an amino group or carboxylic acid, the residue has to have the same atomic number, the same charge, the same number of hydrogen atoms attached, the same chirality and the same bonds to neighbouring atoms. This is not the case for the amino group and carboxylic acid. Here, for each amino group and each carbon atom of a carboxylic acid, any amino or carboxylic acid derivative (as defined in the "Functional groups" section) is a valid match.

There are a some exceptions to this rule: The side chains of *aspartic acid* and *glutamic acid* end with carboxylic acids. Substituting *OH* with $NH_2$ in this group would result in a valid carboxylic acid derivative. But it would also match the structure of *asparagine* / *glutamine*. Therefore, these derivatives are explicitly excluded for aspartic acid and glutamic acid. Note that this does not apply to isopeptide bonds since those use *NH*, not $NH_2$. Similarly, if the amino group of *methionine* is formylated, it becomes *N-formylmethionine*.

In two cases, *arginine* and *histidine*, we allow different tautomers. The tautomeric forms are used by some molecules in ChEBI , e.g., *Ser-Ile-Arg* (CHEBI:163179) and *Ala-His zwitterion* (CHEBI:74388).

It should be stressed that we have been able to obtain the definitions presented in this section only by applying them to thousands of molecules in CHEBI and iteratively testing and correcting the definitions (see the "Development strategy" section). This in turn was possible only via formalising the definitions and making them executable. These tasks will be discussed in the next two sections.

## MSOL Formalisation

This section discusses the monadic second-order logic (MSOL) formalisation of the natural language definitions developed in the section "Defining peptides". We start with a short introduction of MSOL in the section "Monadic second-order logic", followed by a set of general axioms needed for the formalisation (section "General MSOL predicates"). The subsections after that mirror the structure of "Defining peptides", covering classification by charge ("Classification by charge"), the functional groups needed for peptide classification ("Functional groups"), amino acid residues ("Amino acid residues"), peptide structures ("Peptide structures"), specific skeletons ("Structure-based classification") and proteinogenic amino acids ("Presence of proteinogenic amino acids"). Finally, in the section "Proving the taxonomy", we give an outline on how we used MSOL formalisation to provide the taxonomy of the revised peptide ontology.

### Monadic second-order logic

In this paper, MSOL is used to formalise natural language definitions. This formalisation would not be possible in a less expressive logic, e.g., first-order logic (FOL), because the representation of connections between the amino group and carboxyl group of an amino acid requires using the transitive closure of the bond-relation between atoms. Such a transitive closure cannot be expressed in FOL [25, 26]. This can be shown by using compactness (for possibly infinite structures) or Ehrenfeucht-Fraïsse games (for finite structures).

MSOL extends FOL by quantification over monadic, i.e. unary, predicates. Or put differently, MSOL is the restriction of second-order logic that does not allow quantification over arbitrary predicates, but only monadic predicates. For instance, in FOL, one can define *benzene* as a carbon ring of 6 atoms as follows:

1 $$\begin{aligned} \begin{aligned} \text {{Benzene}}(m) \leftrightarrow \exists x_1,&x_2, x_3, x_4, x_5, x_6 (\text {{Molecule}}(m) \wedge \text {{HasPart}}(m, x_1) \\&\wedge \text {{HasPart}}(m, x_2) \wedge \text {{HasPart}}(m, x_3) \wedge \text {{HasPart}}(m, x_4) \\&\wedge \text {{HasPart}}(m, x_5) \wedge \text {{HasPart}}(m, x_6) \\&\wedge C(x_1) \wedge C(x_2) \wedge C(x_3) \wedge C(x_4) \wedge C(x_5) \wedge C(x_6) \\&\wedge \text {{Bond}}(x_1, x_2) \wedge \text {{Bond}}(x_2, x_3) \wedge \text {{Bond}}(x_3, x_4) \\&\wedge \text {{Bond}}(x_4, x_5), \wedge \text {{Bond}}(x_5, x_6) \wedge \text {{Bond}}(x_6, x_1) \\&\bigwedge _{i=1}^5 \bigwedge _{j=i+1}^6 (x_i \ne x_j)) \end{aligned} \end{aligned}$$

Here, the molecule and the atoms are represented by one variable each and we can make statements about each atom and bonds between atoms. However, other classes cannot be defined in this way. For instance, it is not possible to define *cycloalkanes*, i.e., saturated monocyclic hydrocarbons, in FOL. Defining the cycle requires a quantification not over the individual atoms, but over the cycle itself. For this, MSOL is required. In the following formula, *X* is a second-order variable (implicitly quantified universally) and each first-order element *a* (i.e., atom) in *X* has to have exactly 2 bonds to other members of *X* for *X* to be a Cycle. $\text {{IsConnected}}(X)$ means that a all atoms of the cycle have to be connected instead of forming two or more separate circles (cf. Eq. (6) below).

2 $$\begin{aligned} \begin{aligned} \text {Cycle}(X) \leftrightarrow \text {{IsConnected}}(X) \\ \wedge ~ \forall x_1 \in X&\exists x_2, x_3 (\text {{Bond}}(x_1, x_2) \wedge x_2 \in X \\&\wedge ~ \text {{Bond}}(x_1, x_3) \wedge x_3 \in X \wedge x_2 \ne x_3\\&\wedge ~ \forall x_4 ((\text {{Bond}}(x_1, x_4) \wedge x_4 \in X) \rightarrow (x_4 = x_2 \vee x_4 = x_3))) \end{aligned} \end{aligned}$$

Given the Cycle predicate, we can define cycloalkanes as containing only single bonds, exactly one cycle and only carbon atoms. Note that hydrogen atoms are not modelled as elements of the domain. Instead, the number of attached hydrogen atoms is a property of other atoms. Unlike other sources, ChEBI does not allow cycloalkanes with several cycles.

3 $$\begin{aligned} \begin{aligned} \text {{Cycloalkane}}(m) \leftrightarrow&(\text {Molecule}(m) \wedge \forall x_1 (\text {{HasPart}}(m, x_1) \rightarrow (x_1 \in C \\&\quad \wedge \forall x_2 (\text {{Bond}}(x_1, x_2) \rightarrow \text {{SingleBond}}(x_1, x_2)))) \\&\wedge \exists X (\text {{HasPart}}(m, X) \wedge \text {Cycle}(X) \\&\quad \wedge \forall Y ((\text {{HasPart}}(m, Y) \wedge \text {Cycle}(Y)) \rightarrow X = Y))) \end{aligned} \end{aligned}$$

Here, *m*, $x_1$ and $x_2$ are first-order variables while *X* and *Y* are second-order variables. $\text {Molecule}, \text {{HasPart}}, C, \text {{Bond}}, \text {{SingleBond}}$ and $\text {Cycle}$ are predicate names.

More generally, in this work, upper-case letters are used for second-order objects and lower-case letters for first-order objects. Universal quantifiers whose scope is the whole sentence are omitted. Also, set-theoretic notation is used for second-order objects, i.e., $x \in P$ instead of *P*(*x*) and $x \in (P \cup Q)$ instead of $P(x) \vee Q(x)$, $X\subseteq C$ instead of $\forall x (X(x)\rightarrow C(x))$, as well as $R\not =\emptyset$ instead of $\exists x R(x)$ etc.

### Ontology domain vs. single molecule domains

Reasoning about logical entailment in MSOL is undecidable. Hence, in our implementation, we rephrase the problem of classification of molecules as an MSOL model checking problem, which is much more efficient (cf. section "Implementation"). This means that the intended domain of discourse no longer consists of all molecules (and other chemical entities) relevant to ChEBI , but rather of the atoms belonging to a single molecule.

This changes how we formulate definitions. For example, the definition of cycloalkanes (cf. Eq. 3) looks like this in the single molecule domain:

4 $$\begin{aligned} \begin{aligned} \text {{Cycloalkane}} \leftrightarrow&(\forall x_1 (x_1 \in C \wedge \forall x_2 (\text {{Bond}}(x_1, x_2) \rightarrow \text {{SingleBond}}(x_1, x_2))) \\&\wedge \exists X (\text {Cycle}(X) \wedge \forall Y (\text {Cycle}(Y) \rightarrow X = Y))) \end{aligned} \end{aligned}$$

Notably, predicates referring to chemical classes such as *Cycloalkanes* are 0-ary instead of 1-ary. Also, the $\text {Molecule}$ and $\text {{HasPart}}$ predicates are missing as they are not necessary in the single molecule domain. This makes definitions much simpler and more readable.

A limitation of this approach is that one cannot make statements that involve several molecules, for instance when describing reactions. However, this is not needed in the context of this work. Therefore, in the following, we assume single molecule domains.

### General MSOL predicates

Some of the MSOL predicates are not specific to peptides. Therefore, we define them here and refer back to them in the following sections.

Two sets of atoms *overlap* if they share at least one atom:

5 $$\begin{aligned} \text {{HasOverlap}}(X, Y)\leftrightarrow \exists x (x \in X \wedge x \in Y) \end{aligned}$$

A set of atoms is *connected* if it cannot be partitioned into two subsets such that there is no bond between the subsets:

6 $$\begin{aligned} \begin{aligned} \text {{IsConnected}}(X) \leftrightarrow \forall Y, Z (&(Y \ne \emptyset \wedge Z \ne \emptyset \wedge Y \ne Z \wedge Y \cup Z = X) \\&\rightarrow \exists y \in Y, z \in Z \ \text {{Bond}}(y,z)) \end{aligned} \end{aligned}$$

The maximal connected subsets form a partition of the molecule (e.g., the ions of a salt). We refer to these subsets as *connected components*.

7 $$\begin{aligned} \begin{aligned} \text {{ConnectedComponent}}(X) \leftrightarrow (\text {{IsConnected}}(X) \wedge \lnot \exists Y (\text {{IsConnected}}(Y) \wedge X \subset Y)) \end{aligned}\end{aligned}$$

In addition, $\text {{IsConnected}}$ and $\text {{ConnectedComponent}}$ can also be applied to the carbon skeleton of a molecule. This is achieved by limiting $\text {{IsConnected}}$ to carbon atoms.

8 $$\begin{aligned}&\text {{CarbonConnected}}(X) \leftrightarrow (X \subseteq C \wedge \text {{IsConnected}}(X))\\ \end{aligned}$$

The definition of $\text {{CarbonComponent}}$ is the same as for $\text {{ConnectedComponent}}$, but replacing $\text {{IsConnected}}$ with $\text {{CarbonConnected}}$.

9 $$\begin{aligned} \begin{aligned} \text {{CarbonComponent}}(X) \leftrightarrow&(\text {{CarbonConnected}}(X) \\&\wedge \lnot \exists Y (\text {{CarbonConnected}}(Y) \wedge X \subset Y)) \end{aligned}\end{aligned}$$

### Classification by charge

For the charge-based classification, we need to formalise 4 classes: anions, cations, zwitterions and salts. To express charges, we use two types of predicates: $\text {{NetCharge{Positive}}}$, $\text {{NetCharge{Negative}}}$ and $\text {{NetCharge{Neutral}}}$ for the overall charge of a molecule (either greater than 0, lower than 0 or exactly 0) and $\text {{HasCharge{}}}_k$, $\text {{HasCharge{Positive}}}$ and $\text {{HasCharge{Negative}}}$ for a charge of a single atom (either exactly k, greater than 0 or lower than 0). We also use these predicates for sets of atoms (expressing the sum of charges for all atoms in the set). The axioms deriving these second-order predicates from first-order predicates can be found in Appendix "Second-order charge predicates".

Anions and cations are defined by the net charge of the molecule being either negative or positive:

10 $$\begin{aligned} \text {{Anion}} \leftrightarrow (\lnot \text {{Salt}} \wedge \text {{NetCharge{Negative}}}) \end{aligned}$$

11 $$\begin{aligned} \text {{Cation}} \leftrightarrow (\lnot \text {{Salt}} \wedge \text {{NetCharge{Positive}}}) \end{aligned}$$

In addition, salts (defined below) are excluded here explicitly to avoid misclassifications in cases where a salt consists of, for example, 2 anions and 1 cation. While salts are typically neutral, this covers some edge cases such as *fondaparinux sodium* (CHEBI:31632) which are salts (and hence not anions nor cations) but do not have a net neutral charge.

For a zwitterion, the molecule has to have a neutral net charge, but also two connected, but not adjacent atoms of opposite charge:

12 $$\begin{aligned} \begin{aligned} \text {{Zwitterion}} \leftrightarrow \exists X, x_+, x_-&(\text {{NetCharge{Neutral}}} \wedge \text {{IsConnected}}(X) \\&\wedge x_+ \in X \wedge x_- \in X \wedge \text {{HasCharge{Positive}}}(x_+) \\&\wedge \text {{HasCharge{Negative}}}(x_-) \wedge \lnot \text {{Bond}}(x_+, x_-)) \end{aligned}\end{aligned}$$

A salt has an anion and a cation part that are not connected. Here, we can use the notion of connected components introduced in Eq. 6.

13 $$\begin{aligned} \begin{aligned} \text {{Salt}} \leftrightarrow \exists X_+, X_-&(\text {{HasCharge{Positive}}}(X_+) \wedge \text {{HasCharge{Negative}}}(X_-) \\&\wedge \text {{ConnectedComponent}}(X_+) \wedge \text {{ConnectedComponent}}(X_-) \\&\wedge \lnot \text {{HasOverlap}}(X_+, X_-)) \end{aligned}\end{aligned}$$

### Functional groups

Here, we formalise the functional groups discussed in the previous "Functional groups" section, namely carboxylic acid derivatives, amino groups and amide bonds, starting with amide bonds.

Amide bonds are groups of 3 atoms $x_c, x_n$ and $x_o$ that are connected with one single and one double bond. The exact placement of these bonds differs between the amide or imidic form (cf. Fig. 8). For the *OH* group in the imidic form, we explicitly include the deprotonated form $O^-$, which occurs in *peptide zwitterions* and *peptide anions*. The neutral form is defined as having 1 attached hydrogen atom ($\text {{Has{1}Hs}}$) and the deprotonated form is defined as having a charge of -1 ($\text {{HasCharge{M1}}}$).

14 $$\begin{aligned}&\text {{AmideBond}}(x_c, x_o, x_n) \leftrightarrow (x_c \in \text {{C}} \wedge x_o \in \text {{O}} \wedge x_n \in N \\&\wedge ((\text {{SingleBond}}(x_c, x_o) \wedge \text {{DoubleBond}}(x_c, x_n) \wedge (\text {{Has{1}Hs}}(x_o) \vee \text {{HasCharge{M1}}}(x_o))) \\&\quad \vee (\text {{DoubleBond}}(x_c, x_o) \wedge \text {{SingleBond}}(x_c, x_n)))) \end{aligned}$$

Amino groups are nitrogen atoms $x_n$ that only have single bonds and that are only bonded to carbon atoms (hydrogen bonds are not included in the $\text {{Bond}}$-predicate). In addition, we allow non-single bonds if the nitrogen atom is part of an amide bond. Since this goes beyond the definition of amino groups, the term “amino residue” is used instead of “amino group”.

15 $$\begin{aligned} \begin{aligned} \text {{AminoResidue}}(x_n) \leftrightarrow&(x_n \in \text {{N}} \wedge \forall x_c (\text {{Bond}}(x_n, x_c) \rightarrow (x_c \in C \\&\wedge (\text {{SingleBond}}(x_n, x_c) \vee \exists x_o (\text {{AmideBond}}(x, x_o, x_n)) )))) \end{aligned}\end{aligned}$$

In carboxylic acid derivatives ($\text {{CarboxyResidue}}$), three atoms are expected, one of which is the central carbon atom to which an oxygen atom is connected via a double bond and a third atom is connected via a single bond. For the carboxylic acid itself, the third atom is an oxygen atom as well, but in derivatives, it may be substituted by any heteroatom.

16 $$\begin{aligned} \begin{aligned} \text {{CarboxyResidue}}(x_c, x_{double}, x_{single}) \leftrightarrow (x_c \in \text {{C}} \wedge x_{double} \in O \\ \wedge \text {{DoubleBond}}(x_c, x_{double}) \wedge \text {{SingleBond}}(x_c, x_{single})) \end{aligned}\end{aligned}$$

### Amino acid residues

We define amino acid residues in two steps: First, we define the carbon-connected components ($\text {{BuildingBlock}}$). This is done by extending the carbon component defined in Eq. 9 with their direct neighbours. If the neighbour is a nitrogen atom, it cannot be part of an amide bond. Then, we add the heteroatoms to the carbon components, forming complete “building blocks”. Finally, we introduce the presence of carboxylic acid derivatives and amino groups as additional constraints to the building blocks.

17 $$\begin{aligned} \begin{aligned} \text {{BuildingBlock}}(X) \leftrightarrow&\exists Y (\text {{CarbonComponent}}(Y) \wedge Y \subset X \\ \wedge \forall x&(x \in X \rightarrow (x \in Y \vee \exists y (y \in Y \wedge \text {{Bond}}(x,y) \\&\wedge (x \in N \rightarrow \lnot \exists z(\text {{AmideBond}}(y, z, x))))))) \\ \end{aligned}\end{aligned}$$

18 $$\begin{aligned}&\text {{AminoAcidResidue}}(X) \leftrightarrow (\text {{BuildingBlock}}(X) \wedge \exists x_n, x_c, x_{double}, x_{single} \\&\qquad (\text {{AminoResidue}}(x_n) \wedge \text {{CarboxyResidue}}(x_c, x_{double}, x_{single}) \wedge x_n \in X \wedge x_c \in X)) \\ \end{aligned}$$

### Peptide structures

Next, we formalise the peptide structure class in terms of amino acid residues and amide bonds. Since many of the subclasses of peptide structure also rely on the presence of a specific number of amino acids, we define a generalised predicate for "at least n amino acids", of which the general *peptide structure* is a special case for $n = 2$:

19 $$\begin{aligned} \begin{aligned} {\text {PeptideStructure}_{\ge {n}}} \leftrightarrow&\exists X_1, \ldots , X_n, y_{1,1}, y_{1,2}, y_{1,3}, y_{2,1} \ldots , y_{(n-1),3} \\&\bigwedge \limits _{i=1}^n (\text {{AminoAcidResidue}}(X_i) \wedge \bigwedge \limits _{j=i+1}^n \lnot \text {{HasOverlap}}(X_i, X_j)) \\&\wedge \bigwedge \limits _{i=1}^{n-1} \big ((\text {{AmideBond}}(y_{i,1}, y_{i,2}, y_{i,3}) \vee \text {{AmideBond}}(y_{i,3}, y_{i,2}, y_{i,1})) \\&\qquad \qquad \wedge y_{i,1} \in X_{i+1} \wedge \bigvee \limits _{j=1}^i y_{i,3} \in X_{j}\big ) \\ \end{aligned}\end{aligned}$$

This formula defines ${\text {PeptideStructure}_{\ge {n}}}$ as containing *n* amino acid residues (which do not overlap) and $n-1$ amide bonds such that each amide bond connects two amino acids. The restriction that $j \le i$ in the last clause ensures that the resulting structure does not consist of two or more independent chains of amino acids, but one connected structure.

### Classification by size

We can use the ${\text {PeptideStructure}_{\ge {n}}}$ predicates and the charge predicates defined in the section "Classification by charge" to build more complex definitions. Each definition consists of the following parts: A charge description, a lower size bound and (optionally) an upper size bound. The charge description is directly given by the charge predicate (either $\text {{Salt}}, \text {{Zwitterion}}, \text {{Anion}}$ or $\text {{Cation}}$) or, for the neutral case, by a negation of all charge predicates. The lower and upper size bounds are given by a positive and negative ${\text {PeptideStructure}_{\ge {n}}}$ literal, respectively.

For example, *peptide* is defined as follows:

20 $$\begin{aligned} \text {{Peptide}} \leftrightarrow {\text {PeptideStructure}_{\ge {2}}} \wedge \lnot \text {{Salt}}\wedge \lnot \text {{Zwitterion}}\wedge \lnot \text {{Anion}}\wedge \lnot \text {{Cation}} \end{aligned}$$

Here, no upper bound is given as all chains of at least 2 amino acids are peptides. An example with upper bound are *Tripeptide zwitterions*, which are zwitterions and have at least 3 amino acids, but less than 4 amino acids:

21 $$\begin{aligned} \text {{TripeptideZwitterion}} \leftrightarrow \text {{Zwitterion}} \wedge {\text {PeptideStructure}_{\ge {3}}} \wedge \lnot {\text {PeptideStructure}_{\ge {4}}} \end{aligned}$$

### Structure-based classification

Formally, we define both classes in this category (*2,5-diketopiperazines* and *emericellamides*) directly by their superclasses and skeletons. This formalisation is very lengthy. The full definitions in TPTP format can be found in Additional File 2.

### Presence of proteinogenic amino acids

The identification of specific proteinogenic amino acids builds upon the existing definition of amino and carboxy residues (Eqs. 15 and 16). These are used in places where (theoretically) an amide bond or some other modification can change the structure of the original amino acid. In all other places, we describe each atom individually via 3 predicates: One for the element (e.g., carbon), one for the charge and one for the number of attached hydrogen atoms. The bonds between atoms are specified as well. Additionally, the chirality of the alpha carbon atom is given (except for glycine) to differentiate L-amino acids from non-proteinogenic D-amino acids.

For example, *L-alanine residue* is defined as follows:

22 $$\begin{aligned}&\text {{LAlanineResidue}}(a_n, a_\alpha , a_c, a_1) \leftrightarrow (\text {{AminoResidue}}(a_n) \wedge \text {{SingleBond}}(a_n, a_\alpha ) \\&\quad \wedge \text {{HasCharge{0}}}(a_\alpha ) \wedge a_\alpha \in C \wedge \text {{Has{1}Hs}}(a_\alpha ) \\&\quad \wedge \exists a_s, a_d \ \text {{CarboxyResidue}}(a_c, a_s, a_d) \\&\quad \wedge \text {{SingleBond}}(a_\alpha , a_c) \wedge \text {{SingleBond}}(a_\alpha , a_1) \\&\quad \wedge \lnot \exists x (\text {{Bond}}(a_\alpha , x) \wedge x \ne a_n \wedge x \ne a_c \wedge x \ne a_1) \\&\quad \wedge a_1 \in C \wedge \text {{HasCharge{0}}}(a_1) \wedge \text {{Chiral{S}}}(a_\alpha ) \wedge \text {{Has{3}Hs}}(a_1)) \end{aligned}$$

The definitions for all 23 proteinogenic amino acids in TPTP format can be found in Additional File 3.

### Proving the taxonomy

Given the MSOL definitions discussed in this section, we can prove that the subsumption relations of the revised peptide ontology (cf. section "Overview of revised peptide ontology") hold. In some cases, this is trivial. For instance, by definition, a molecule is a *dipeptide* if and only if it is a *dipeptide structure* and a *peptide*. For more complex relations, namely those of the size-based taxonomy, the subsumption relations have been proven using the theorem prover cvc5 [27].

## Implementation

The main challenge with MSOL is reasoning about logical entailment, i.e. about the question whether a logical formula (called consequence) is entailed by a set of logical axioms (i.e. true in all models of the axioms). For proving subsumptions between classes that are logically entailed by their defined in MSOL, we need full MSOL reasoning, which is undecidable in general. Yet, we have proven some subsumption relations in the class hierarchy (cf. section "Proving the taxonomy").

Luckily, due to the single molecule domain approach introduced in the section "Ontology domain vs. single molecule domains", for the classification of molecules, we need to tackle a much easier problem, namely MSOL model checking. This is the problem to answer the question whether a model (representing a certain molecule) satisfies a set of axioms (specifying a certain class of molecules). While MSOL model checking is decidable and is much easier than reasoning, it still is of high computational complexity. More specifically, it is PSPACE-complete in the size of the model [28] (linear in case of bounded tree-width [29]), and non-elementary in the size of the formula [30].

Therefore, for the automatic classification of peptides, we have taken three measures which, in combination, make automatic classification computationally feasible while staying as close as possible to the original definitions.

The first measure has already been discussed in "Ontology domain vs. single molecule domains". Instead of performing reasoning over the whole ontology (as would be usual for an OWL ontology), we make use of the fact that the classification of each molecule can be treated independently from other molecules.

The second measure is the translation of a monadic second-order problem into a first-order one. This step is discussed below (cf. sections "Model Checking" and "Model checking in first-order logic"). Finally, an algorithmic classification has been developed that can be verified using the FOL axiomatisation (cf. "Algorithmic implementation").

The implementation is available on GitHub [31] Also, a web interface in which users can classify their own molecules and get justifications for specific classification results is available [16]. A more detailed explanation of our methodology for translating MSOL definitions into FOL and algorithmic implementations can be found in [32].

The results reported in this section and "Evaluation" refer to an evaluation carried out on a AMD EPYC 7742 processor with 512MB of memory and no GPU.

### Model checking

Given our MSOL axiomatisation of classes, classification of molecules amounts to a model checking problem. To this end, a molecule is represented as a FOL structure. For example, *Met-*$\beta$*-ala* from Fig. 4 is represented by the following structure:

23 $$\begin{aligned} \begin{aligned} D^\mathfrak {M} =&\{c_1, c_2, c_3, c_4, c_5, c_6, c_7, c_8, o_1, o_2, o_3, n_1, n_2, s\} \\ \mathfrak {M}(\text {{C}}) =&\{c_1, c_2, c_3, c_4, c_5, c_6, c_7, c_8\} \\ \mathfrak {M}(\text {{N}}) =&\{n_1, n_2\} \\ \mathfrak {M}(\text {{O}}) =& \{o_1, o_2, o_3\} \\ \mathfrak {M}(\text {{S}}) =& \{s\} \\ \mathfrak {M}(\text {{Has{0}Hs}}) =&\{c_5, c_8, o_1, o_2, s\} \\ \mathfrak {M}(\text {{Has{1}Hs}}) =& \{c_4, o_3, n_2\} \\ \mathfrak {M}(\text {{Has{2}Hs}}) =&\{c_2, c_3, c_6, c_7, n_1\} \\ \mathfrak {M}(\text {{Has{3}Hs}}) =& \{c_1\} \\ \mathfrak {M}(\text {{HasCharge{0}}}) =&D^\mathfrak {M}, \mathfrak {M}(\text {{NetCharge{)}}} =& 0 \\ \mathfrak {M}(\text {{Bond}}) =&\{(c_1, s_1), (s_1, c_1), (s_1, c_2), (c_2, s_1), \ldots \} \\ \mathfrak {M}(\text {{SingleBond}}) =&\{(c_1, s_1), (s_1, c_1), (s_1, c_2), (c_2, s_1),\ldots \} \\ \mathfrak {M}(\text {{DoubleBond}}) =&\{(c_5, o_1), (o_1, c_5), (c_8, o_2), (c_8, o_2),\ldots \} \end{aligned}\end{aligned}$$

The question is then: Does this structure satisfy an axiomatisation of a given class? If yes, then the molecule belongs to that class.

### Model finding with MONA

We start with using the MSOL axiomatisation directly, despite the high complexity of MSOL model checking. A problem is that no MSOL model checker is available, which is why we have to use a MSOL model finder, MONA [33], instead. Model finding means to find a model that satisfies a given set of axioms. Model finding has a large search space (all possible models) and is much harder than model checking, where the model is given in advance, and only a formula needs to be evaluated in that model.

Using a model finder for model checking sounds like using a sledgehammer to crack a nut—after all, MSOL model checking is decidable, while MSOL model finding is not. However, we will encode our given model with an MSOL formula, which reduces the search space for the model finder dramatically, and essentially leaves MONA with a model checking problem. As a note to logicians, we also want to stress that a crucial part of MSOL model checking for formulas containing existential quantifiers over sets is to find specific witness sets for these quantifiers. This is equivalent to finding extensions of unary predicates in a (restricted) model finding problem, where the existentially quantified variables have been replaced by (Skolem) unary constant predicates.

A MONA model finding problem consists of 3 components:

1. Molecule description: Axioms characterising the FOL model representing the input molecule (e.g., stating that atom 4 is a nitrogen atom with one attached hydrogen). Properties include the element, charge and attached hydrogen atoms for every heteroatom, as well as the bond type for every bond.
2. Background axioms: The intermediate definitions that have been formulated in section 4.
3. Target axioms: This is the definition of the final class.

As an example, to prove that *Met-*$\beta$*-ala* (cf. Fig. 4) is a *peptide structure*, the following problem has to be satisfiable:

24 $$\begin{aligned} \begin{aligned} \text {{Atom}}=&\{c_1, c_2, c_3, c_4, c_5, c_6, c_7, c_8, o_1, o_2, o_3, n_1, n_2, s\} \\ \text {{C}} =&\{c_1, c_2, c_3, c_4, c_5, c_6, c_7, c_8\} \\ \text {{O}} =&\{o_1, o_2, o_3\}, \text {{N}} = \{n_1, n_2\} \\ \text {{S}} =& \{s\}, \\ \text {{Has{0}Hs}} = &\{c_5, c_8, o_1, o_2, s\} \\ \text {{Has{1}Hs}} =& \{c_4, o_3, n_2\} \\ \text {{Has{2}Hs}} =&\{c_2, c_3, c_6, c_7, n_1\} \\ \text {{Has{3}Hs}} =& \{c_1\} \\ \text {{HasCharge{0}}} =&\text {{Atom}}, \\ \text {{NetCharge {=}}} &\ 0 \\ \text {{SingleBond}}(x, y) \leftrightarrow&((x = c_1 \wedge y = s) \vee (x = s \wedge y \in \{c_1, c_2\}) \vee \ldots ) \\ \text {{DoubleBond}}(x, y) \leftrightarrow&((x = c_5 \wedge y = o_1) \vee (x = o_1 \vee y = c_5) \vee \ldots ) \\ \text {{Bond}}(x, y) \leftrightarrow&(\text {{SingleBond}}(x, y) \vee \text {{DoubleBond}}(x, y)) \\ \text {{HasOverlap}}(X, Y) \leftrightarrow&(\exists z (z \in X \wedge z \in Y)) \\ \text {{IsConnected}}(X) \leftrightarrow&\ldots , \text {{ConnectedComponent}}(X) \leftrightarrow \ldots \\ \text {{CarbonComponent}}(X)&\leftrightarrow \ldots , \text {{AmideBond}}(a_c, a_o, a_n) \leftrightarrow \ldots \\ \text {{AminoResidue}}(a_n)&\leftrightarrow \ldots , \text {{CarboxyResidue}}(a_c, a_{double}, a_{single}) \leftrightarrow \ldots \\ \text {{BuildingBlock}}(X) \leftrightarrow&\ldots , \text {{AminoAcidResidue}}(X) \leftrightarrow \ldots \\ \exists A_0, A_1, b_{1,1}, b_{1,2}, b_{1,3}&(\text {{AminoAcidResidue}}(A_0) \wedge ~\text {{HasOverlap}}(A_0, A_1) \\&\wedge \text {{AminoAcidResidue}}(A_1) \wedge b_{1,1} \in A_1 \wedge b_{1,3} \in A_0 \\&\wedge (\text {{AmideBond}}(b_{1,1}, b_{1,2}, b_{1,3}) \vee \text {{AmideBond}}(b_{1,1}, b_{1,2}, b_{1,3}))) \end{aligned}\end{aligned}$$

We have classified the 1,000 shortest peptides (measured by their SMILES length) with MONA. For more than half (545), this returned a result in an average time of 9.5 s. MONA failed to classify the remaining ones due to not-configurable memory constraints. This failure rate increases for larger molecules.

The successfully classified peptides have been used to identify potential errors in the FOL-based classification.

### QBF satisfiability

The MSOL model checking problem can also be encoded in Quantified Boolean Formulas (QBF) Here, the predicate extensions are represented with propositional variables. For example, if the atom *a* is in the extension of the predicate *C*, we add a positive literal $C_a$ to the theory. If *a* is not in the extension, the negative literal $\lnot C_a$ is used. Quantification over first-order variables is represented by conjunctions (for universal quantification) and disjunctions (for existential quantification) indexed over all atoms in the domain. Quantification over second-order variables turns into quantification over *n* boolean variables where *n* is the size of the domain (each boolean variable is true exactly if the corresponding atom belongs to the second-order variable).

QBF problems are converted to CNF using a Tseitin transformation [34], preprocessed with Bloqqer [35] and solved by DepQBF [36]. In our implementation, MONA and QBF problems (in the QDIMACS format) are automatically generated from an abstract MSOL representation.

### Model checking in first-order logic

As shown in the section "MSOL formalisation", our definition of peptides cannot be expressed in FOL. This is due to the transitive closure of the bond relation which is needed in two places: First, when differentiating salts and zwitterions, connected components of the molecule graph have to be identified. And secondly, the definition of amino acids requires defining a chain of carbon atoms.

Therefore, when defining amino acids in FOL, one can only make statements about amino acids in terms of the carbon-connected building blocks, not define the building blocks themselves:

25 $$\begin{aligned} \begin{aligned} \text {{AminoAcidResidue}}(x) \leftrightarrow&(\text {{BuildingBlock}}(x) \\ \wedge \exists a, c_1, c_2, c_3&(\text {{AminoResidue}}(a) \wedge \text {{CarboxyResidue}}(c_1, c_2, c_3) \\&\wedge \text {{HasOverlap}}(a, x) \wedge \text {{HasOverlap}}(c_1, x) \\&\wedge \text {{HasOverlap}}(c_2, x) \wedge \text {{HasOverlap}}(c_3, x))) \end{aligned}\end{aligned}$$

In contrast to MSOL, here, $\text {{BuildingBlock}}$ and $\text {{HasOverlap}}$ are not logically defined. Instead, they are primitive predicates, whose extensions are computed algorithmically, and get added to the FOL model introduced in the "Model checking" section.

A crucial difference to the MSOL formalisation is that $\text {{BuildingBlock}}$ is no longer applied to a second-order variable *X*, but rather to a first-order variable *x*. The reason is that in FOL, we simulate quantification over sets as follows: Given a FOL model, we reify sets of atoms as new elements of its universe. As a result, we can use first-order variables for both atoms and sets of atoms. When quantifying, we have to restrict variables to atoms or sets, respectively. However, often this happens implicitly by the use of predicate. E.g., $\text {{BuildingBlock}}$ is defined to hold only for sets, which implies that in the above formula, we do not need to express explicitly that *x* is a set.

For FOL, we have implemented a model checker with which we can classify molecules. This has been tested on the 3-star subset of ChEBI with 45,450 molecules (including non-peptides). The classification failed for $2.2\%$ of instances, either due to memory limitations or after reaching the timeout of 30 s. For the remaining instances, the average time per instance was 1.03 seconds. This includes multiple model checking runs (for charge-based, size-based, substructure-based and amino acid classification). Compared to MSOL model finding, it constitutes a speed up by a factor of nearly 10, with significantly less failures and on a broader dataset.

The results from this evaluation, as well as a classification of the remaining ChEBI (i.e., the 2-STAR subset) have been used to verify the algorithmic implementation (cf. section "Algorithmic implementation").

### Algorithmic implementation

In the section "Model checking in first-order logic", the idea of replacing certain logical definitions with algorithmically calculated extensions has been introduced. Here, we take this idea one step further. While FOL model checking is already relatively efficient, there is still potential for improvement. This leads us to a fully algorithmic classification, effectively replacing all logically defined predicates with algorithmically defined ones.

In practice, the algorithmic classification uses functions from the cheminformatics library RDKit [37] that are also used in the construction of axiomatic representations of molecules and FOL structures. For some tasks, a graph representation of the molecule is used, speeding up the classification with graph algorithms from the networkx [38] package. Additionally, the side chains of proteinogenic amino acids are specified using SMARTS expressions.

To compare the performance of the algorithmic implementation to FOL model checking, we ran it on ChEBI ’s 3-STAR subset as well. We obtained an average time per molecule of 8.4 milliseconds without any timeouts. This makes it a highly reliable and scalable method, outperforming FOL model checking by a factor of more than 100. At the same time, we observed no differences in the classification results between MSOL model finding, QBF satisfiability, FOL model checking and the algorithmic implementation. This can be expected since most translations have been performed automatically. Only the conversion of predicate definitions that require MSOL to an algorithmic implementation was conducted manually. Also, the results from each step have been used in the development process for the subsequent steps.

To demonstrate the scalability of the algorithmic classification, we have applied it to 119.3 million molecules from PubChem. Here, the average time per molecule was slightly higher at 1.7 milliseconds. Also, 70 molecules could not be classified due to reaching a size limit of 10,000 carbon-connected components. This limit has been set since larger molecules have infeasibly long computation times. Nevertheless, this shows that it is possible to apply the peptide classification rules to large datasets. The full classification results on PubChem are available in Additional File 4.

## Evaluation

In this section, we evaluate the quality of our classification. First, we investigate quantitatively how our classification behaves for ChEBI molecules (cf. section "Quantitative evaluation"). Also, we want to find out how well our classification aligns with ChEBI . This is done by a comparison with existing ChEBI labels (cf. "Qualitative comparison with ChEBI"). Since there are no classes in ChEBI for peptides that are made out of specific proteinogenic amino acids, we use different sources of information for this part of the evaluation (cf. "Proteinogenic amino acids"). Finally, in order to get more insights into the accuracy and limitations of our classification, a survey with domain experts has been conducted on molecules which are classified differently by us compared to ChEBI . The results of this survey are discussed in the section "Expert survey". The full classification results of our approach on ChEBI molecules can be found in Additional File 5.

In all evaluations, we employ a flattened version of the class hierarchy. This means that, when discussing molecules classified as *oligopeptide* (either by ChEBI or by our approach), we consider all molecules classified as oligopeptides, including both molecules that are directly classified as oligopeptides and molecules that are indirectly classified as oligopeptides (and also belong to one of the subclasses of oligopeptide such as dipeptide).

### Quantitative evaluation

**Table 2:** Average number of assigned classes by method

			Excluding negative samples	
Classification	Full ChEBI	3-star subset	Full ChEBI	3-star subset
Ours (67 classes)	0.73	0.37	6.79	6.95
Ours (14 classes)	0.29	0.13	2.75	2.42
ChEBI (14 classes)	0.12	0.08	1.13	1.54

The 67 classes are all classes in our revised peptide ontology, the 14 the ones shared with ChEBI. In the right-hand columns, molecules that were not assigned to any class by any method have been excluded

For the evaluation, we have classified all ChEBI molecules (i.e., SMILES-annotated classes) with our methodology. On average, our classification is able to assign 0.73 classes to each molecule (cf. Table 2). In practice this means that most molecules (89.5%) receive no classes while every molecule with a peptide structure gets at least 4 classes (peptide structure, a charge-based and size-based subclass as well as the combination of size- and charge-based classes). This is to be expected since ChEBI covers a broad range of biologically relevant classes and is not limited to peptides. On average, molecules that are assigned at least one class by either our classification or ChEBI get assigned 6.79 classes. At the maximum, three molecules (*exendin-3*, CHEBI:75469; *exendin-4*, CHEBI:64073 and *lixisenatide*, CHEBI:85662) have been assigned to 22 classes each since they are made up of 18 different proteinogenic amino acids each.

When taking only the 14 classes into account that are shared between the revised ontology and ChEBI, our approach assigns more than double the amount of classes (0.29 / 2.75 excluding negative samples) compared to ChEBI (0.12 / 1.13). This effect is less pronounced on molecules from the 3-star subset, but our methodology still assigns both more classes overall and, for the shared classes, more molecules per class. In general, the class assignments per molecule are lower on the 3-star subset (in our classification, 94.8% of 3-star molecules have received no class). This can be attributed to most peptides in ChEBI belonging to the 2-star subset. Accounting for that, ChEBI makes slightly more classifications per molecule in the 3-star subset (1.54 to 1.13), which can be expected since the 3-star subset is manually curated and therefore less likely to have incomplete class assignments.

### Qualitative comparison with ChEBI

In this section, we compare our classification against ChEBI’s in more detail, with the following questions: Where, how often and why does our classification get in conflict with ChEBI? And where do we extend the existing classification?

**Table 3:** Comparison between our approach and ChEBI for the shared classes

ChEBI class	Total	Ours		**ChEBI**		Neither	Cohen’s kappa
		abs	rel	abs	rel		
60194 (peptide cation)	167	167	1.000	42	0.251	178,948	0.402
60334 (peptide anion)	298	298	1.000	100	0.336	178,817	0.502
60466 (p. zwitterion)	137	135	0.985	84	0.613	178,978	0.749
90799 (dip. zwitterion)	105	103	0.981	60	0.571	179,010	0.712
155837 (trip. zwitterion)	15	15	1.000	8	0.533	179,100	0.696
16670 (peptide)	18,507	18,122	0.979	14,750	0.797	160,608	0.861
25676 (oligopeptide)	17,278	17,106	0.990	4,502	0.261	161,837	0.376
46761 (dipeptide)	4,911	4,793	0.976	1,212	0.247	174,204	0.357
47923 (tripeptide)	9,089	9,075	0.998	219	0.024	170,026	0.042
48030 (tetrapeptide)	710	704	0.992	134	0.189	178,405	0.305
48545 (pentapeptide)	722	718	0.994	20	0.028	178,393	0.043
15841 (polypeptide)	1,100	1,016	0.924	468	0.425	178,015	0.516
64372 (emericellamide)	10	10	1.000	6	0.600	179,105	0.750
65061 (2,5-diketopip.)	339	310	0.914	68	0.201	178,776	0.206
Sum	53,388	52,572	0.985	21,673	0.406	2,454,222	0.556
Mean	3,813	3,755	0.981	2,167	0.363	175,302	0.465

Total: The number of ChEBI molecules assigned to the class by either our approach or ChEBI. Ours / ChEBI absolute: The number of molecules assigned to the class by our approach / ChEBI. Ours / ChEBI relative: The absolute value divided by Total. Neither: The number of molecules not assigned to the class by either method. The sum row shows the sums of the absolute values and the relative scores calculated over the sums. The mean row calculates the mean of each column independently

For each of the 14 classes that are shared between our revised ontology and ChEBI, we can analyse how our classification differs from ChEBI. The results can be seen in Table 3. For each class, the “Total” column counts the number molecules that are assigned to the class either by us or by ChEBI, “Neither” counts the molecules that are not assigned to this class by either method. “Ours” / “ChEBI” count how many molecules were assigned to a class by one method (either in absolute terms or relative to “Total”). Cohen’s kappa [39] measures the agreement between both approaches and is calculated as

26 $$\begin{aligned} \kappa = \frac{2 \times (\text {Both} \times \text {Neither} + (\text {Both}-\text {ChEBI}) \times (\text {Ours} - \text {Both}))}{\text {Ours} \times (\text {Ours} - \text {Both} + \text {Neither}) + \text {ChEBI} \times (\text {ChEBI}-\text {Both} + \text {Neither})} \end{aligned}$$

where

27 $$\begin{aligned} \text {Both} = \text {Ours} + \text {ChEBI} - \text {Total}. \end{aligned}$$

A derivation of this formula can be found in Appendix "Cohen’s Kappa".

Overall, our method makes about 53 thousand positive classifications while ChEBI makes 22 thousand positive classifications. Out of all positive classifications only about 800 or 1.5% have not been made by our method and solely by ChEBI. In contrast, 59.4% of positive classification have only been made by our method. The overlap between both methods consists of 20,857 positive classifications that ChEBI and our method agree on. For the negative classifications, there are 2.45 million cases in which a molecule has not been assigned to a class by either method. This is mainly caused by the large number of non-peptides, since ChEBI has a far broader scope than peptides.

Comparing different classes, the agreement (i.e., Cohen’s kappa) varies drastically. While Cohen’s kappa for the peptide class lies at 86%, it is only 4% for tripeptides and pentapeptides. This can be traced back to the large number of additional molecules assigned to these classes by our approach.

**Table 4:** Comparison between our approach and ChEBI on ChEBI’s 3-star subset

ChEBI class	Total	Ours		**ChEBI**		Neither	Cohen’s kappa
		abs	rel	abs	rel		
60194 (peptide cation)	58	58	1.000	32	0.552	45,392	0.711
60334 (peptide anion)	240	240	1.000	88	0.367	45,210	0.535
60466 (p. zwitterion)	117	115	0.983	76	0.650	45,333	0.774
90799 (dip. zwitterion)	91	89	0.978	55	0.604	45,359	0.736
155837 (trip. zwitterion)	14	14	1.000	8	0.571	45,436	0.727
16670 (peptide)	1,894	1,860	0.982	1,416	0.748	43,556	0.838
25676 (oligopeptide)	1,682	1,650	0.981	981	0.583	43,768	0.714
46761 (dipeptide)	916	902	0.985	570	0.622	44,534	0.752
47923 (tripeptide)	399	387	0.970	194	0.486	45,051	0.624
48030 (tetrapeptide)	152	146	0.961	119	0.783	45,298	0.852
48545 (pentapeptide)	63	61	0.968	18	0.286	45,387	0.405
15841 (polypeptide)	210	210	1.000	99	0.471	45,240	0.640
64372 (emericellamide)	6	6	1.000	6	1.000	45,444	1.000
65061 (2,5-diketopip.)	28	27	0.964	22	0.786	45,422	0.857
**Sum**	5,870	5,765	0.982	3,684	0.628	630,430	0.756
**Mean**	419	443	0.984	409	0.608	45,031	0.726

Total: The number of 3-star molecules assigned to the class by either our approach or ChEBI. Ours / ChEBI absolute: The number of molecules assigned to the class by our approach / ChEBI. Ours / ChEBI relative: The absolute value divided by Total. Neither: The number of molecules not assigned to the class by either method. The sum row shows the sums of the absolute values and the relative scores calculated over the sums. The mean row calculates the mean of each column independently

To understand where this disagreement comes from, we make the same comparison for molecules from ChEBI’s 3-star subset (cf. Table 4). Here, the agreement is much higher for most classes, with an average Cohen’s kappa of 73% compared to 47% on the full ChEBI.

The difference between the full ChEBI and the 3-star subset can be seen most clearly for *tripeptides*. While there are 8,870 molecules that are tripeptides by our classification (but not by ChEBI), most of them are in the 2-star subset and only 205 are in the 3-star subset. Most of these molecules follow the same naming pattern: *Abc-Def-Ghi*, where *Abc*, *Def* and *Ghi* are 3-letter abbreviations for proteinogenic amino acids. Using this pattern, we have identified 7,993 molecules in ChEBI. Out of those, 136 belong to the 3-star subset and are subclasses of tripeptide. In the 2-star subset, the most common direct superclasses are peptide (5,899 molecules) and oligopeptide (1,926). Only 16 molecules are subclasses of tripeptide. As of May 2025, this issue has been addressed in ChEBI. This indicates that, for large parts of the 2-star subset of ChEBI , the ChEBI classification does not necessarily conflict with our classification. Instead, we have identified missing axioms in ChEBI.

**Fig. 12:**
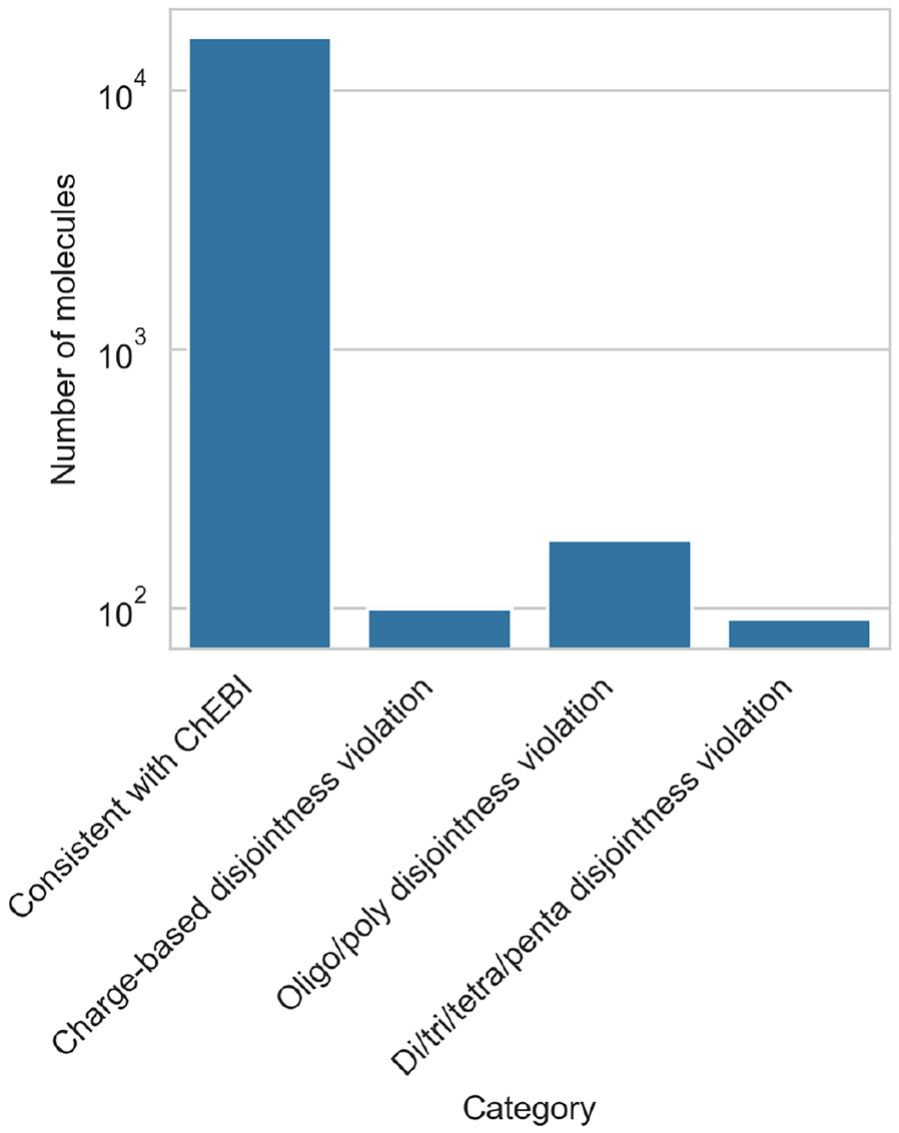
Inconsistencies with ChEBI for molecules where we assigned at least one new class

To further investigate to which degree our classification is an extension or a contradiction to ChEBI, the 16,327 molecules (2-star and 3-star) where we assigned at least one new class have been analysed more closely. Our classification is *inconsistent* with ChEBI if any of the newly-assigned classes are disjoint with any ChEBI-assigned class. We consider the disjointness relations shown in Fig. 2. In result, 371 (2.27%) of the analysed molecules have a classification inconsistent with ChEBI (cf. Fig. 12). This can be broken down into 99 molecules that have a different charge-based classification (peptide, peptide anion, cation and zwitterion are disjoint), 182 molecules that were oligopeptides in ChEBI but polypeptides in our classification (or vice versa) and 90 molecules that have a different subclassification within the oligopeptide class (violating the pairwise disjointness between di-, tri-, tetra- and pentapeptide).

**Fig. 13:**
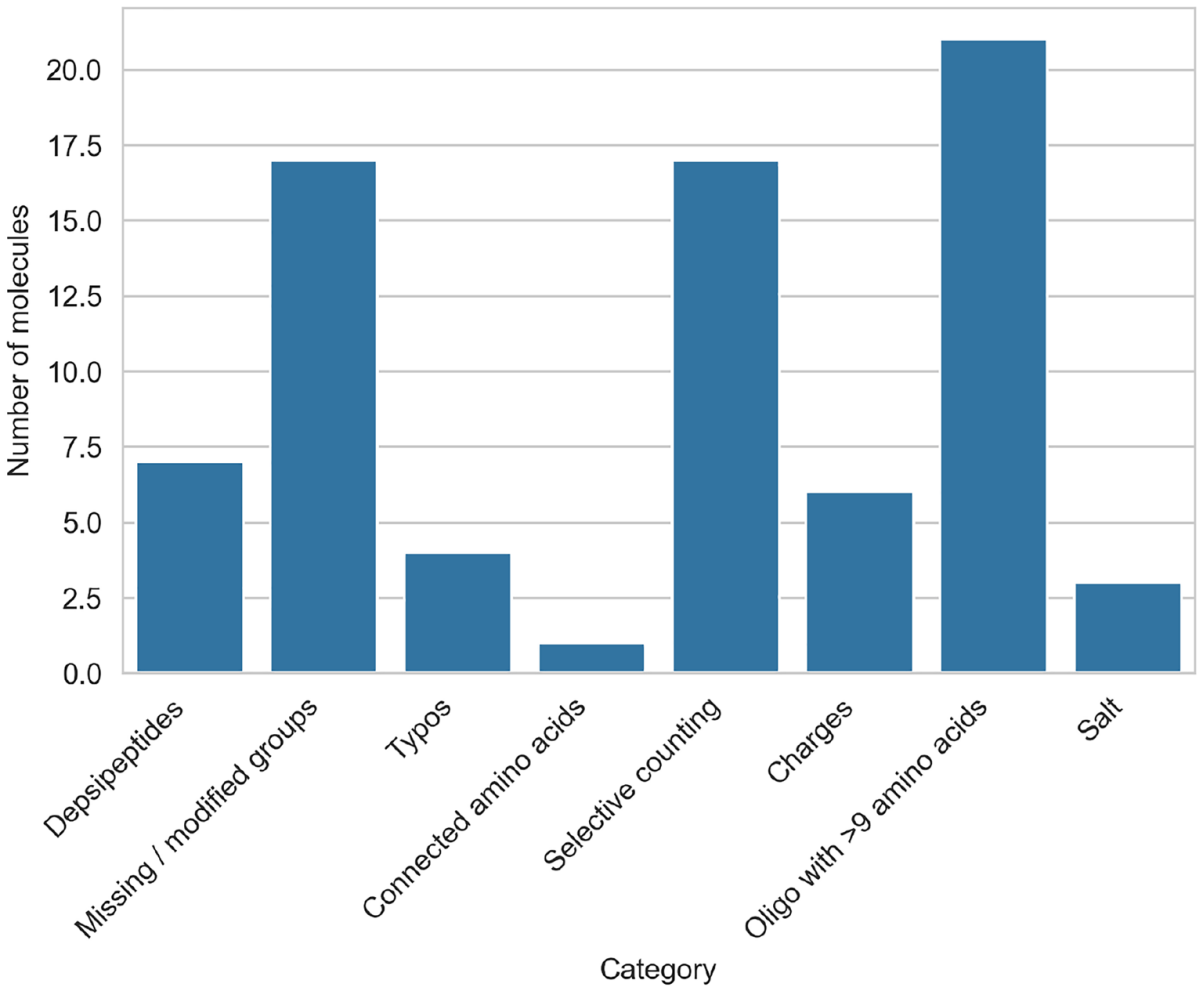
Manually assigned categories for molecules from the 3-star subset where ChEBI made a classification that has not been made by our approach

For a closer examination, we have focused on the 3-star subset. Of particular interest here are the 76 molecules that were explicitly assigned to a class in ChEBI which has not been assigned by us. This includes 50 molecules in which ChEBI and our classification are inconsistent. We have manually assessed and categorised these molecules. Here, we give only an overview (see Fig. 13) and some examples. A complete list of the molecules with explanations can be found in Additional File 6.

The most common type of mismatch are **oligopeptides with more than 9 amino acids**. As discussed in section 3, we have decided to treat all molecules with at least 10 amino acids as polypeptides and not as oligopeptides. However, ChEBI does not mention a clear limit for the number of amino acids and uses different limits in practice. Therefore, this mismatch can be expected.

Another large category are molecules with **missing or modified groups**. Here, one of the functional groups constituting a peptide has been replaced or modified such that it does not fulfil our definition. Still, they bear similarity to peptides or are synthesised from peptides, explaining the assigned ChEBI class. For instance, if *glutaurine* (CHEBI:27694) had a carboxylic acid instead of a sulfonic acid, it would fulfil our definition.

The third category captures a process we call **selective counting**. In some cases, a peptide has both proteinogenic and non-proteinogenic amino acids. Then, some classes in ChEBI seemingly apply a narrow definition of amino acid, taking only the proteinogenic ones into account. Take for example $\beta$*-Ala-Lys-*$N^\epsilon$*-AMCA* (CHEBI:79309). This molecule has 3 components that are connected by amide bonds. 2 of them, $\beta$*-alanine* and *lysine*, are clearly labelled as proteinogenic amino acids. The third, *(7-amino-4-methylcoumarin-3-yl)acetic acid* (AMCA), is not labelled as an amino acid, justifying the classification as a dipeptide. Since we take all components as amino acids that have an amino group and a carboxylic acid derivative, we come to the conclusion that the molecule should be classified as a tripeptide instead.

**Charges** and **Salt** refer to molecules that are not neutral, but still classified as peptides. **Depsipeptides** are members of a peptide subclass in ChEBI where hydroxy acids and amino acids are used instead of only amino acids. If this sequence is alternating (an amino acid residue is connected to a hydroxy acid residue), then there are no two consecutive amino acids and the molecule does not fulfil our definition of peptides.

Finally, *mycocyclosin* (CHEBI:71596) is a special case where the side chains of a dipeptide have been **connected**. In most cases where two amino groups are connected via a chain of carbon atoms, they belong to the same amino acid. Therefore, for the sake of consistency, we have decided to keep this rule, even though it leads to this unintuitive result.

In addition to the 3-star molecules, polypeptides and 2,5-diketopiperazines from the 2-star subset have been analysed as well. These classes are particularly interesting as there are nearly no conflicts for the 3-star molecules. Nevertheless, in the 2-star subset, there are 84 polypeptides and 29 2,5-diketopiperazines in ChEBI that our classification was not able to reproduce. The only such case in the 3-star subset is *mycoclosin* which has been discussed above.

For the *polypeptide* class, the recurring pattern involved molecules that clearly have fewer than 10 amino acid residues (e.g., *myxarylin*, CHEBI:222409 with 2 peptide bonds). Thereby, they form the counterpart to oligopeptides with more than 9 amino acids. Most likely, some of the externally sourced molecules do not follow ChEBI’s definition that polypeptides have to have at least 10 amino acid residues. For the class *2,5-diketopiperazines*, we have found modifications of the *piperazine-2,5-dione* skeleton that are not allowed by the our definition, such as additional disulfide bridges (as in *A26771A*, CHEBI:203748) or anionic forms (as in *7-dimethylallyl isoechinulin B anion*, CHEBI:193014).

### Proteinogenic amino acids

A large part of our classification, the classification by residues of proteinogenic amino acids, is not related to any existing ChEBI classes. However, in many cases, ChEBI still documents which amino acids make up a given molecule. In the following, we will distinguish different sources of information.

One source are *has functional parent* OWL axioms in ChEBI. These are used to describe that a molecule derived by functional modification. In both class labels and definitions, we have performed a text search for amino acid names. These names come in 3 different forms: Either as 3-letter abbreviations, e.g. *Ala*, as the full name, e.g. *alanine*, or as the full name for the proteinogenic enantiomer, e.g. *L-alanine*. This results in a total of 7 ways to determine if an amino acid is related to a given peptide.

**Table 5:** Comparison between our classification according to proteinogenic amino acids against different information sources in ChEBI

ChEBI information source	Total	Ours		ChEBI		Neither	Cohen’s kappa
		abs	rel	abs	rel		
has functional parent	38,303	38,294	1.000	992	0.026	395,155	0.046
abbreviation in name	40,393	38,294	0.948	25,659	0.635	393,065	0.717
amino acid in name	38,533	38,294	0.994	455	0.012	394,925	0.009
enantiomer name in name	38,331	38,294	0.999	186	0.005	395,127	0.007
abbreviation in definition	38,411	38,294	0.997	1,101	0.029	395,047	0.045
amino acid in definition	38,677	38,294	0.990	2,259	0.058	394,781	0.084
enantiomer name in def	38,361	38,294	0.998	1,792	0.047	395,097	0.079

The table counts all pairs of ChEBI molecules which are peptide structures (following our definition) and proteinogenic amino acids. Total: The number of molecule-amino acid pairs where either our approach has detected a residue of the amino acid in the molecule or where the ChEBI information source suggests a connection between the molecule and the amino acid. Ours / ChEBI absolute: The number of pairs according to our approach / ChEBI. Ours / ChEBI relative: The absolute value divided by Total. Neither: The number of pairs where the molecule and amino acid were not connected by either method

We have compared our classification to each information source (taking each information source as substitute for a class) for the 23 proteinogenic amino acids. The results can be seen in Table 5. For all information sources, there are few peptide-amino acid pairs that were not found by our classification (1% or less, except for *abbreviation in name*). Also, the ChEBI information is relatively sparse compared to our classification. The by far most extensive information source is *abbreviation in name* with 26 thousand cases. Therefore, it also has the highest Cohen’s kappa with 0.72.

For the peptide-amino acid pairs which are suggested by an information source but have not been found with our classification, we have performed a closer analysis. With the *has functional parent* relation, 9 cases are classified positively that we classify negatively. There, the original amino acid has been modified in other ways than through the formation of peptide bonds. For instance, *Ac-Tyr-Val-Ala-Asp-chloromethylketone* (CHEBI:230455) has a relation *has functional parent* *some* *L-aspartic acid*. However, the carboxyl group of *L-aspartic acid* has been substituted by a chloromethyl group. This shows that the *functional parent* relation is broader than the one we are looking for when identifying the amino acids constituting a peptide.

For the other information sources, there are more missed molecule-amino acid pairs, although differences exist: For instance, only in 37 cases, peptides have an enantiomer name in their name without the amino acid being identified by our approach. If we look for the amino acid name without enantiomer information (e.g., “alanine” instead of “L-alanine”), this number grows to 239 cases. Mostly, these are amino acids with a D configuration. Therefore, these cases are not a contradiction to our classification which is specific to L-amino acids.

An illustrative example of the limitations of the ChEBI information source we use is *apelin-12* (CHEBI:149669). It comes up for the *enantiomer name in definition* information sources because the definition states that apelin-12 is “A 12 amino acid oligopeptide fragment of apelin-13 lacking the terminal L-phenylalanine residue.” Here, it is apparent that our classification is correct in not finding L-phenylalanine in the molecule.

In some rare cases, the mismatches between our classification and the extracted labels were due to errors in the ontology. We have reported those errors and they have subsequently been fixed.

### Expert survey

In the section "Qualitative comparison with ChEBI", we have identified and categorised molecules where our classification contradicts the ChEBI classification. To get a better understanding of these molecules, we have selected 11 molecules which are representative of reoccurring patterns we have observed during manual inspection of our results. For 9 of these molecules, the classifications were contradictory. 2 molecules where our classification agrees with ChEBI were included as well to gather insights into the different viewpoints on these molecules. All 11 molecules have been compiled into a questionnaire alongside additional explanations for both our and the ChEBI classifications and have been given independently to 3 domain experts. For each molecule, we have asked the experts if they agree with our classification or ChEBI (possibly both or none) and to give a short explanation. The questionnaire can be found in the Additional Files 7 and 8.

**Fig. 14:**
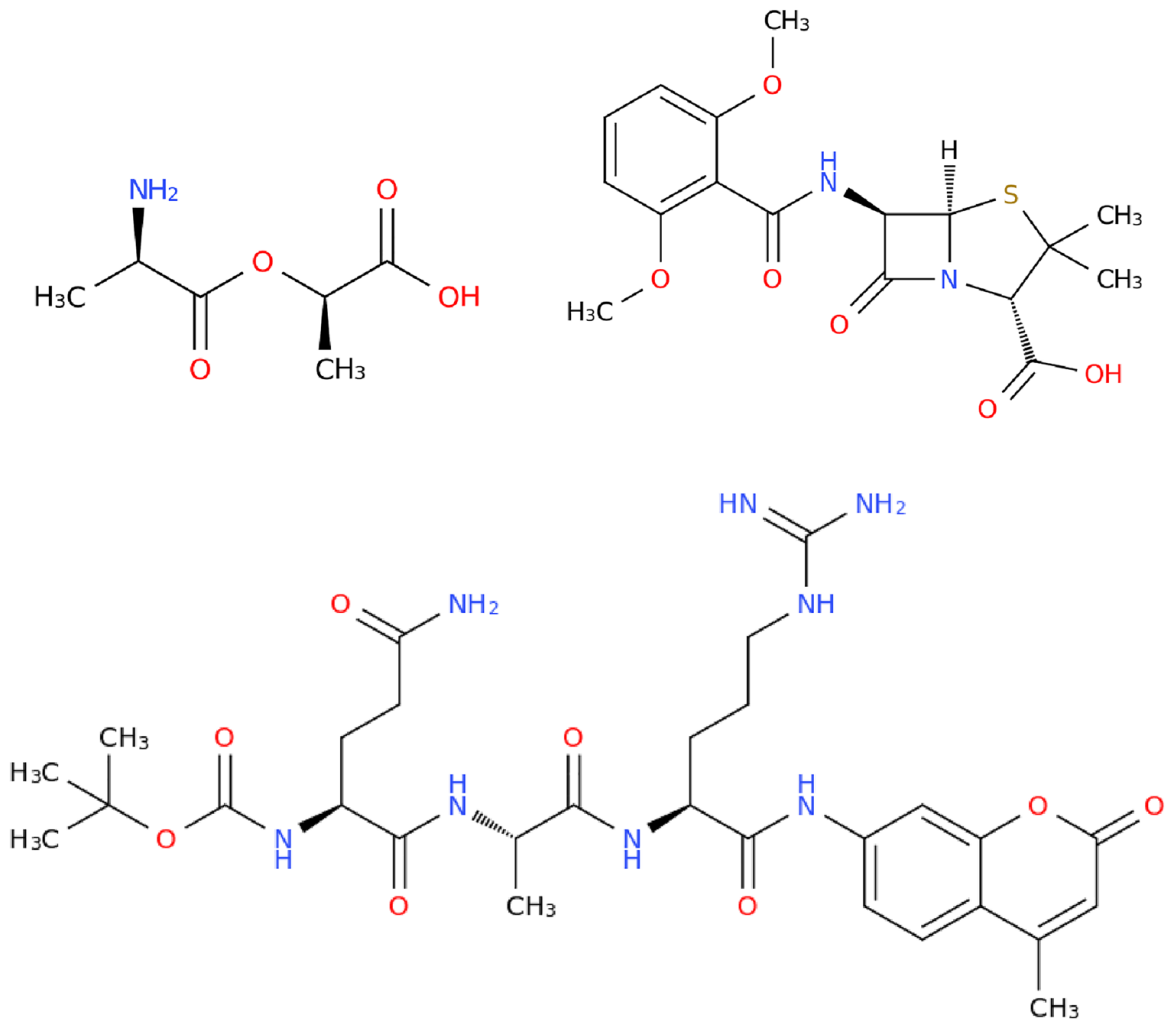
3 molecules from the expert survey: *D-alanyl-(R)-lactic acid* (CHEBI:61163, top left), *Methicillin* (CHEBI:6827, top right) and *Boc-Gln-Ala-Arg-7-amino-4-methylcoumarin* (CHEBI:84832).

Overall, the results were mixed. On average, each expert agreed with ChEBI for 5.67 molecules and with our classification for 6 molecules. While there is a consensus for most molecules, the experts expressed diverging opinions for 3 molecules. In the following, we will discuss 3 examples from the survey which are shown in Fig. 14.

#### D-alanyl-(R)-lactic acid (CHEBI:61163)

*D-alanyl-(R)-lactic acid* (CHEBI:61163) has been chosen for the questionnaire because it is classified by ChEBI not only as a peptide, but as a *depsipeptide*. Depsipeptides are a class of molecules which consist of a sequence of amino and hydroxy carboxylic acid residues. For *D-alanyl-(R)-lactic acid*, we come to the result that it is not a peptide. All experts agreed with our classification. Nevertheless, it is still worth discussing the exact reasons given by the expers to understand where the difference between our classification and ChEBI stems from.

One reviewer stated that “strictly adhering to IUPAC definitions, the molecule is a depsipeptide, but not a peptide.”. Classifying a molecule as only a depsipeptide would not be possible in ChEBI , because depsipeptide is a subclass of peptide. Another expert argued that *D-alanyl-(R)-lactic acid* is not a depsipeptide altogether, and that amide groups are a necessary component of depsipeptides. Here, the problem is not the relation between the depsipeptide and peptide classes, but between the molecule and the depsipeptide class. The third questionee did not give a definitive answer on whether *D-alanyl-(R)-lactic acid* is a depsipeptide.

#### Methicillin (CHEBI:6827)

*Meticillin* belongs to the class of *penicillins*, which all share the same basic structure. Due to this structure, they are classified as peptides in our classification system. ChEBI however does not see them as peptides. Two of the experts agreed that *meticillin* is a peptide, citing that the molecule can be split into two amino acid residues along the sulfur atom and the central amide bond. This is the same step that is performed by our classification, which separates amino acid residues at arbitrary heteroatoms. In contrast, the third expert does not consider this as an option, instead arguing that, because the hydrolysation of only the peptide bond does not yield two separate fragments, the molecule does not constitute a peptide.

#### Boc-Gln-Ala-Arg-7-amino-4-methylcoumarin (CHEBI:84832)

This molecule has unanimously been classified as a tripeptide by the questionees. Thus, they agree with the ChEBI classification, while our classification claims four amino acid residues. The reason for this is that the amino-coumarin at the C-terminus is identified as an amino acid residue. More specifically, the experts disagree with the assessment that coumarin contains a carboxylic acid residue. Instead, they point out that the “carboxy moiety is part of a ring structure in the form of a cyclic ester / lactone” and that “coumarins do not exist in an open carboxylic acid form”. This makes the classification of amino-coumarin as an amino acid residue a clear misclassification. Our definitions do not take ring structures into consideration although they are relevant for classification in this case.

## Discussion

In section Evaluation, the peptide class definitions have been evaluated in two ways: First, by a comparison with ChEBI (sections "Quantitative evaluation, Qualitative comparison with ChEBI and Proteinogenic amino acids") and secondly, by a manual categorisation in the section "Qualitative comparison with ChEBI"and with an "Expert survey".

The "Quantitative Evaluation" has shown that the classification with our revised ontology is more fine-grained than the ChEBI classification. Out of the 67 classes we propose, only 14 correspond to ChEBI classes. This allows us to differentiate peptide-like molecules better, assigning 6.79 classes on average compared to ChEBI, which only assigns 1.13. For instance, a molecule like $\beta$*-alanyl-L-arginium* (CHEBI:143157) has one peptide-related superclass in ChEBI (peptide cation), but 7 in our revised ontology (peptide structure, peptide cation, oligopeptide structure, dipeptide structure, oligopeptide cation, dipeptide cation, arginine peptide structure). While some of the classes are trivial, we capture two pieces of information that ChEBI only implies by the textual definition without stating them formally in OWL: The molecule consists of two amino acid residues and one of them is a residue of L-arginine.

In addition to adding new classes, we also make full use of the existing ChEBI classes: For the 14 shared classes, we make more than twice the number of classifications on average. This is mostly due to the *oligopeptide* class and its subclasses (cf. Table 3). For instance, *Z-Arg-Arg* (CHEBI:10094) is only classified as a peptide in ChEBI, which is correct but incomplete. According to our classification, it is an oligo- and dipeptide as well.

Another aspect that has been evaluated is the quality of our predictions. Out of all molecules where we assigned at least one new class, 97.73% are consistent with the ChEBI classification (cf. Fig. 12). This means that, in most cases, we either confirm the ChEBI classification or extend it in a non-conflicting way.

A manual evaluation of molecules where conflicts did arise has shown some reoccurring patterns (cf. Fig. 13). While some conflicts are clearly mistakes in the ChEBI classification, such as *glutaurine* (this has been confirmed to be a mistake by the expert survey), most can be traced back to ambiguities in class definitions: Is a molecule with 10 amino acid residues still an oligopeptide? Does a dipeptide consist of 2 amino acids or 2 proteinogenic amino acids? Are depsipeptides peptides? And after which modifications does a peptide remain a peptide or become a non-peptide?

All of these questions may be answered differently by experts from different domains, and have been interpreted differently even among ChEBI annotators. This has been confirmed by our "Expert survey" where, in some cases, the questionees came to different conclusions for the same molecules. Our approach forces us to set clear rules and helps identifying how molecules would be affected by decisions. In the development process, molecule classifications that are different to ChEBI have been used to identify potential gaps in our definitions. Therefore, we do not assume that our definition of peptides is the only correct one. Instead, it is one of several plausible ones that may be modified in one way or another.

In the section "Proteinogenic amino acids", we have evaluated the detection of proteinogenic amino acids in peptides. While this connection is important for many users, indicated by the common usage of amino acid names and abbreviations in the class labels and definitions, there is no OWL object property in ChEBI that captures this relation exactly. A close approximation is *has functional parent*, which ChEBI uses in some cases to relate peptides to the amino acids from which they were formed. Our evaluation in Table 5 has shown that our approach is able to identify these relations correctly, deducing 9 molecules where, according to manual inspection, *has functional parent* refers to other modifications than the formation of peptide bonds. This, as well as our comparison against class labels and definitions, gives us reasonable evidence that the detected peptide-amino acid relations are consistent with ChEBI. In total, we have identified more than 38,000 peptide-amino acid relations, showing the potential for an extension of ChEBI.

## Conclusion

In this work, a set of definitions for peptides and peptide-related classes has been proposed. For a total of 67 concepts, natural language definitions have been developed. These concepts and definitions are based on the peptide subhierarchy of the ChEBI ontology. It has been discussed how our definitions extend the existing definitions in ChEBI, resolving ambiguities and covering cases that cannot be easily decided with the current definition.

We have provided a formalisation of the proposed definitions in MSOL. Using the methodology developed in [32], the MSOL definitions have been translated into FOL and an algorithmic implementation. In an iterative development process, the natural language definitions have been stated more precisely, taking the molecules already present in ChEBI into account.

The result is not only a formalised, improved definition of peptides, but an automatic classification tool. This has made it possible to evaluate how well the developed classification corresponds to the ChEBI molecules. In the vast majority of cases, the classification tool was able to reproduce existing classifications while extending them with additional subclasses. For instance, many of the molecules which were only assigned to *peptide* have been identified to be *tripeptides*.

The molecules where our classification does not align with ChEBI have been analysed more closely. Some of them have been evaluated independently by 3 experts. While they agreed on 8 of the 11 evaluated molecules, they arrived at different conclusions for the remaining 3. This shows that, while the definition of peptides itself is not controversial, the exact interpretation is not fixed. For other molecules, we were able to identify errors or inconsistencies in ChEBI.

In future work, we will expand our methodology to other parts of the chemical domain. This includes further automating the translation steps from MSOL to FOL that cannot yet be performed automatically.

Also, it will require an analysis of the definition of ChEBI classes: While some classes are not defined by their structure at all (e.g., *peptide antibiotic*), different types of structural classification are possible as well. In this work, we have already seen three: Classification by charge (e.g., peptide anions vs. peptide cations), by size (e.g., oligo- vs. polypeptides) and by specific substructures (e.g., 2,5-diketopiperazines). Our goal is to build a comprehensive framework that can use and combine different classification types.

Another line of research will be the combination of the formal logic-based approach with stochastic models in an ensemble approach. Here, the idea is that some classes can be classified well via formal methods while others can be covered by different types of machine learning models.

## Figures and Tables

**Fig. 15:**
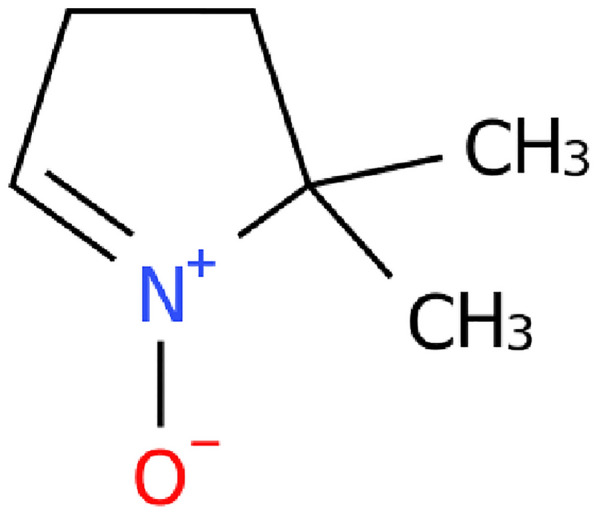
*5,5-dimethyl-1-pyrroline N-oxide* (CHEBI:149489) has both a positive and a negative charge

## Data Availability

The source code for the peptide classification is available on GitHub: https://github.com/sfluegel05/chemlog-peptides. The web interface for peptide classification can be found https://chebifier.hastingslab.org. The source code for our web interface is available at https://github.com/ChEB-AI/Chebifier. Supplementary data is available on Zenodo at https://doi.org/10.5281/zenodo.16566621.

## Second-order charge predicates

In the context of salts, we have used the predicates $\text {{HasCharge{Positive}}}$ and $\text {{HasCharge{Negative}}}$ for second-order elements.

Here, we give a formal definition of these predicates. The main complication with these definitions is that they depend on some form of counting. While the charge of single atoms is directly given by the model, the charge of a set of atom has to be inferred. Typically, one would take all charges of atoms in the set and sum them up. However, counting with and comparing arbitrary numbers would require an extension of MSOL, like MSOL with cardinality constraints [40].

Our definition works by assuming a maximum value $k_{max}$ for the number of positive or negative charges in a molecule. For a given molecule, $k_{max}$ is bounded by the number of atoms and the maximum absolute charge of an atom. This allows us to express $\text {{HasCharge{Positive}}}$ and $\text {{HasCharge{Negative}}}$ via a disjunction of $\text {{ContainsPositiveCharges}}_{\ge k}$ and $\text {{ContainsNegativeCharges}}_{\ge k}$ predicates, denoting a positive or negative charge of at least *k*:

28 $$\begin{aligned} \begin{aligned} \text {{HasCharge{Positive}}}(X) \leftrightarrow \bigvee _{k=1}^{k_{max}}&(\text {{ContainsPositiveCharges}}_{\ge k}(X) \\&\wedge \lnot \text {{ContainsNegativeCharges}}_{\ge k}(X)) \end{aligned}\end{aligned}$$

29 $$\begin{aligned} \begin{aligned} \text {{HasCharge{Negative}}}(X) \leftrightarrow \bigvee _{k=1}^{k_{max}}&(\text {{ContainsNegativeCharges}}_{\ge k}(X) \\&\wedge \lnot \text {{ContainsPositiveCharges}}_{\ge k}(X)) \end{aligned}\end{aligned}$$

Note that $\text {{ContainsPositiveCharges}}_{\ge k}$ and $\text {{ContainsNegativeCharges}}_{\ge k}$ do not refer to the sum of all charges of a set of atoms, but only to the sums of positive and negative charges respectively. For instance, the atoms of *5,5-dimethyl-1-pyrroline N-oxide* (Fig. 15) would fulfil both $\text {{ContainsPositiveCharges}}_{\ge 1}$ (due to the positive nitrogen atom) and $\text {{ContainsNegativeCharges}}_{\ge 1}$ (due to the negative oxygen atom).

For a given *k*, $\text {{ContainsPositiveCharges}}_{\ge k}$ and $\text {{ContainsNegativeCharges}}_{\ge k}$ are defined via disjunction of all possible combinations of atoms leading to a charge of at least *k*. For instance, for $\text {{ContainsPositiveCharges}}_{\ge 2}$, there are two options: Either at least two atoms with a single positive charge each or one atom with at least two positive charges.

Formally, this is expressed as

30 $$\begin{aligned} \begin{aligned} \text {{ContainsPositiveCharges}}_{\ge k}(A) \leftrightarrow&\bigvee _{(x_1, x_2, \ldots , x_n) \in \mathcal {P}(k)} \exists a_1, a_2, \ldots , a_n \\&\bigwedge _{i=1}^n (a_i \in A \wedge \text {{HasCharge{}}}_{x_i}(a_i) \wedge \bigwedge _{j=1}^{i-1} a_i \ne a_j) \end{aligned}\end{aligned}$$

and

31 $$\begin{aligned} \begin{aligned} \text {{ContainsNegativeCharges}}_{\ge k}(A) \leftrightarrow&\bigvee _{(x_1, x_2, \ldots , x_n) \in \mathcal {P}(k)} \exists a_1, a_2, \ldots , a_n \\&\bigwedge _{i=1}^n (a_i \in A \wedge \text {{HasCharge{}}}_{-x_i}(a_i) \wedge \bigwedge _{j=1}^{i-1} a_i \ne a_j) \end{aligned}\end{aligned}$$

where

32 $$\begin{aligned} \begin{aligned} \mathcal {P}(k) = \{(x_1, x_2, \ldots , x_n) \in \mathbb {N}_+^n | \sum _{i=1}^n x_i = k, x_1 \ge x_2 \ge \ldots \ge x_n\} \end{aligned}\end{aligned}$$

Note that, by definition, $n \le k$ for all $(x_1, x_2, \ldots , x_n) \in \mathcal {P}(k)$ since every $x_i$ has to be positive. This limits the size of $\mathcal {P}(k)$ for a given *k*.

## Cohen’s Kappa

Cohen’s kappa [39] is a common statistic for measuring inter-rater agreement [41]. It is generally defined as

33 $$\begin{aligned} \kappa = \frac{P_o - P_e}{1 - P_e} \end{aligned}$$

where $P_o$ is the probability of the raters agreeing and $P_e$ the probability of the raters agreeing by chance.

In the context of this work, the raters are our re-classification and the ChEBI classification of molecules. A separate kappa is calculated for each class, giving both raters the options of either a positive or a negative classification. In section 6.2, molecule classifications are sorted into 4 categories: **Total** (*t*) for all molecules that are assigned to the given class by either our classification or ChEBI , **Ours** (*o*) for molecules assigned to the class by our classification, **ChEBI ** (*c*) if the molecule is assigned to the class by ChEBI and **Neither** (*n*) for molecules not assigned to the class at all. Note that the first 3 categories are overlapping, while Neither captures exactly the molecules that do not belong to the first 3 categories. For simplicity, we will also use **Both** (*b*), defined as

34 $$\begin{aligned} b = o + c - t \end{aligned}$$

In the following, we define Cohen’s kappa in terms of these 5 categories. $P_o$ is set to the number of molecules where both raters yield the same results, divided by the total number of molecules. The same result occurs if both methods make a negative classification or if both make a positive classification:

35 $$\begin{aligned} P_o = \frac{b + n}{t + n} \end{aligned}$$

$P_e$ measures chance agreement. Therefore, we need to calculate the probability of both raters making a positive classification ($P_p$) and the probability of both raters making a negative classification ($P_n$):

36 $$\begin{aligned} P_p = \frac{o}{t + n} \times \frac{c}{t + n} = \frac{o \times c}{(t + n)^2} \end{aligned}$$

37 $$\begin{aligned} P_n = \frac{n + c - b}{t + n} \times \frac{n + o - b}{t + n} = \frac{(n + c - b) \times (n + o - b)}{(t+n)^2} \end{aligned}$$

38 $$\begin{aligned} P_e = P_p + P_n \end{aligned}$$

Inserting this into the equation for Cohen’s kappa, we get

39 $$\begin{aligned} \begin{aligned} \kappa&= \frac{P_o - P_p - P_n}{1 - P_p - P_n} \\&= \frac{\frac{b + n}{t + n} - \frac{oc}{(t + n)^2} - \frac{(n + c - b)(n + o - b)}{(t+n)^2}}{1 - \frac{oc}{(t + n)^2} - \frac{(n + c - b)(n + o - b)}{(t+n)^2}} \\&= \frac{(b+n)(t+n)-oc-(n+c-b)(n+o-b)}{(t+n)^2-oc-(n+c-b)(n+o-b)} \\ \end{aligned} \end{aligned}$$

Inserting $t = o + c - b$, this resolves to

40 $$\begin{aligned} \begin{aligned} \kappa&= \frac{(b+n)(o+c+n-b)-oc-(n+c-b)(n+o-b)}{(o+c+n-b)^2-oc-(n+c-b)(n+o-b)} \\&= \frac{2(bo + bc + bn - b^2 - oc)}{o^2+on-ob+c^2+cn-cb} \\&= \frac{2(bn+(b-o)(c-b))}{o(o+n-b)+c(c+n-b)} \end{aligned} \end{aligned}$$

which is the form of $\kappa$ used in our evaluation.

## Overview of predicate names

Table 6 gives a comprehensive overview of MSOL predicate names used throughout the paper. The arities are given for single molecule domains (cf. section Ontology domain vs. single molecule domains). The arguments of a predicate can be either first-order (FO) or second-order (SO) objects. Not included are predicates that are only used as examples (e.g. Benzene in section Monadic second-order logic). We distinguish between two types of predicates: (1) Predicates whose extensions can be directly derived from the given molecule and are therefore part of the FOL structure (cf. section Model checking). (2) Predicates which are defined in terms of other predicates. Here, we reference the definition for each predicate.

**Table 6:** Predicate names used throughout this paper.

Name	Arity	Source	Description
C, O, N, $\ldots$	1 (FO)	FOL structure	Atom symbols
HasChargeM1, HasCharge0, HasCharge1, .	1 (FO)	FOL structure	Atom charge
Has0Hs, Has1Hs, $\ldots$	1 (FO)	FOL structure	Number of attached hydrogen atoms
Bond, SingleBond, DoubleBond	2 (FO)	FOL structure	Covalent bonds
NetCharge	0	FOL structure	Charge of a molecule
HasOverlap	2 (SO)	Eq. 5	Two sets of atoms that share at least one atom
IsConnected	1(SO)	Eq. 6	Set of connected atoms
ConnectedComponent	1 (SO)	Eq. 7	Largest possible set of connected atoms
CarbonConncted	1 (SO)	Eq. 8	Set of connected carbon atoms
CarbonComponent	1 (SO)	Eq. 9	Largest possible set of connected carbon atoms
Anion	0	Eq. 10	
Cation	0	Eq. 11	
Zwitterion	0	Eq. 12	
Salt	0	Eq. 13	
AmideBond	3 (FO)	Eq. 14	
AminoResidue	1 (FO)	Eq. 15	
CarboxyResidue	3 (FO)	Eq. 16	
BuildingBlock	1 (SO)	Eq. 17	Backbone of an amino acid residue
$\text {{AminoAcidResidue}}$	1 (SO)	Eq. 18	
${\text {PeptideStructure}_{\ge {n}}}$	0	Eq.19	Peptide structure of at least *n* amino acids
Peptide, PeptideZwitterion, $\ldots$	0	Eq. 20	Subclasses of peptide structure with specified charge category
Tripeptide, TripeptideZwitterion, $\ldots$	0	Eq. 21	Subclasses of peptide structure with specified charge and amino acid count
Diketopiperazines	8 (FO)	Additional File 2	Structure-based peptide subclass
Emericellamide	34 (FO)	Additional File 2	Structure-based peptide subclass
LAlanineResidue, $\ldots$	varying	Eq. 22, Additional File 3	Residues of proteinogenic amino acids

## Footnotes

1 The subsequent discussion is based on ChEBI version 239. We intend to submit the results of this work as improvement to ChEBI, such that eventually, the limitations of ChEBI described here will disappear.

2 To our knowledge, *lepirudin* (CHEBI:142437) is the largest peptide in ChEBI with 65 amino acids.
